# Nutritional intake of *Aplanochytrium* (Labyrinthulea, Stramenopiles) from living diatoms revealed by culture experiments suggesting the new prey–predator interactions in the grazing food web of the marine ecosystem

**DOI:** 10.1371/journal.pone.0208941

**Published:** 2019-01-09

**Authors:** Yoko Hamamoto, Daiske Honda

**Affiliations:** 1 Graduate School of Natural Science, Konan University, Okamoto, Higashinada, Kobe, Hyogo, Japan; 2 Institute for Integrative Neurobiology, Konan University, Okamoto, Higashinada, Kobe, Hyogo, Japan; 3 Department of Biology, Faculty of Science and Engineering, Konan University, Okamoto, Higashinada, Kobe, Hyogo, Japan; Stazione Zoologica Anton Dohrn, ITALY

## Abstract

Labyrinthuleans (Labyrinthulea, Stramenopiles) are recognized as decomposers in marine ecosystems but their nutrient sources are not fully understood. We conducted two-membered culture experiments with labyrinthuleans and diatoms to discover where labyrinthuleans obtain their nutrients from. The results showed that *Aplanochytrium* strains obtained nutrients by consuming living diatoms. *Aplanochytrium* cells did not release digestive enzymes into the medium, but adhered to diatom cells via the tip of their characteristic ectoplasmic net system to obtain nutrients from them. The chloroplast and cell contents of the diatoms shrank and were absorbed, and then the number of *Aplanochytrium* cells rapidly increased as multiple aplanospores were released. To estimate the effect of labyrinthulean organisms including *Aplanochytrium* on marine ecosystem, we explored the dataset generated by the Tara Oceans Project from a wide range of oceanic regions. The average proportion of all labyrinthulean sequences to diatom sequences at each station was about 10%, and labyrinthulids, oblongichytrids, and aplanochytrids were the major constituent genera, accounting for more than 80% of labyrinthuleans. Therefore, these groups are suggested to greatly affect the marine ecosystem. There were positive correlations between aplanochytrids and phototrophs, green algae, and diatoms. At many stations, relatively large proportions of aplanochytrid sequences were detected in the size fraction larger than their cell size. This implied that *Aplanochytrium* cells increased their particle size by adhering to each other and forming aggregates with diatoms that are captured by larger zooplankton in the environment, thereby bypassing the food web pathway via aplanochytrids to higher predators. The intake of nutrients from diatoms by aplanochytrids represents a newly recognized pathway in the grazing food chain in the marine ecosystem.

## Introduction

The class Labyrinthulea is a group in the Stramenopiles and are ubiquitous marine heterotrophs [1–7]. This group is characterized by the presence of the ectoplasmic net system, which is produced by a unique organelle known as the bothrosome [2, 8–12]. The Labyrinthulea comprises at least four phylogenetically distinct groups [2, 5, 13–17]: the labyrinthulids are classified as *Labyrinthula* spp., whose spindle-shaped cells are located in the ectoplasmic net element; the thraustochytrids account for almost all genera whose spherical cells have rhizoid-like ectoplasmic nets produced from a single bothrosome; the oblongichytrids are classified as *Oblongichytrium* spp., which are characterized by slender oblong zoospores; and the aplanochytrids are classified as *Aplanochytrium* spp., which form hexagonal scaly walled sporangia that release non-flagellate gliding cells known as aplanospores.

The labyrinthuleans secrete degrading enzymes from (e.g. cellulase, protease, and lipase), and absorb nutrients into, the characteristic ectoplasmic net system, so they are recognized as important decomposers in marine ecosystems [18–24]. Many are concentrated on dead seaweeds and seagrasses, but *Labyrinthula zosterae* and Quahog Parasite Unknown (QPX, a thaustochyrid) are able to infect seaweeds and mollusks (see [25–28]). Field studies have shown that comparatively large numbers of labyrinthuleans exist in river discharge areas, and that labyrinthulean abundance is correlated with particulate and dissolved organic carbon (POC and DOC, respectively) but not chlorophyll *a* [29–31]. Thus, it has been suggested that most labyrinthuleans at river mouths consume non-phytoplankton-derived POC and DOC such as terrestrial organic matter and marine detritus.

Another study suggested that habitat segregation of labyrinthuleans occurs between river mouth and coastal areas, because continuous monitoring over several years revealed that the composition of genera clearly differed between the two areas [31]. Their results showed that *Schizochytrium* sp. and *Oblongichytrium* spp. tended to occur at the river mouth, *Aplanochytrium* sp. was more typical of coastal areas; and one lineage of in the genus *Oblongichytrium* was present in both areas. In that monitoring study, the labyrinthuleans were cultured and isolated from environmental samples to estimate cell numbers and detect phylogenetic groups. That method tends to underestimate cell numbers, because certain labyrinthuleans are not detected if they do not grow on agar media with antibiotics [31, 32]. The genera *Aplanochytrium* and *Oblongichytrium* do not well grow on agar media. Despite their poor growth on agar media, these genera are recognized as being abundant among labyrinthuleans, and even more abundant than estimated from culturing analyses.

Recently, the genus *Aplanochytrium* has been suggested to be ecologically important. Aplanochytrids have been detected from geographically widespread areas including the Arabian Sea [33], the 0–1000 m water column in the equatorial Indian Ocean [34], the Ross Sea and the Antarctic Ocean [35, 36], and coastal areas of the western Pacific Ocean [31]. Aplanochytrids have also been isolated from the gut and fecal pellets of zooplankton [34, 37–38]. Metagenomic analyses revealed a higher proportion of aplanochytrids in the gut contents of copepods than in environmental samples [39], suggesting that aplanochytrids are associated with zooplankton in predator–prey or commensal relationships. In addition, it was reported that *Aplanochytrium* cells secrete cellulase, so it suggests aplanochytrids play a role as a decomposer for plant substances [22]. In contrast, 80% of 18S rDNA phylogenetic lineages detected by sequencing labyrinthulid-specific amplicons aligned with aplanochytrids in Hawaiian water [40]. Similarly, sequences of aplanochytrids and oblongichytrids were mainly detected among labyrinthulid-specific amplicons from seawater and sediment samples [41]. Aplanochytrids have commonly been detected by metagenomic analyses in sea water, and not only from coastal areas [42–43]. Moreover, 18S rDNA sequences clustered with aplanochytrid sequences in the phylogenetic trees have been recognized in universal 18S rDNA libraries constructed from sediment and seawater samples from extreme environments such as deep sea and hydrothermal vents (e.g., [44–49]).

As mentioned above, aplanochytrids are cosmopolitan and abundant heterotrophs that interact with zooplankton, so they may have important ecological roles that have been overlooked so far. However, the aplanochytrids, like other labyrinthuleans, were cultured in the artificial media containing organic matter in the most previous studies, because they have been convinced as decomposers targeting organic matter such as marine detritus (e.g., [14, 31]). So, the nutrient sources of aplanochytrids have never been determined. Therefore, the role and impacts of aplanochytrids in the marine ecosystem are unknown. In this study, we discovered that the aplanochytrids actively consume nutrients from living diatoms and then increase their biomass before reproducing in two-membered culture experiments. Diatoms are the major primary producers in coastal areas, so this discovery suggests the new prey-predator interactions in the grazing food web of the marine ecosystem.

## Materials and methods

### Culture maintenance

Labyrinthulean strains were maintained in liquid culture medium, d-GPY, consisting of 0.05% w/v yeast extract, 0.1% w/v poly-peptone, and 0.2% w/v glucose in 1:1 seawater/distilled water. The diatom strains were maintained in f/2 medium [50]. All the media were sterilized in an autoclave. Table 1 shows the incubation temperature and sampling site for each strain. We selected *Skeletonema* and *Chaetoceros* strains to grow in two-membered cultures with labyrinthuleans, because these genera are cosmopolitan and ubiquitous diatoms in the oceans, and their biomasses are large [51]. All examined strains were axenic cultures.

**Table 1:** Labyrinthulean and diatom strains used in the study.

Strain number	Taxon	Temperature (°C)	Sampling site	Reference
Labyrinthuleans				
KMPB N-BA-107	*Aplanochytrium kerguelense*	25	South Indian Ocean	[3]
SEK 602	*Aplanochytrium* sp.	20	Osaka Bay, Japan	[31]
SEK 717	*Aplanochytrium* sp.	25	Osaka Bay, Japan	[31]
SEK 754	*Aplanochytrium* sp.	25	Osaka Bay, Japan	[31]
SEK 758	*Aplanochytrium* sp.	25	Osaka Bay, Japan	[31]
NBRC 110806	*Aurantiochytrium* sp.	25	Shukugawa River mouth, Hyogo, Japan	[31]
NIBH N1-27	*Aurantiochytrium* sp.	25	West Pacific Ocean, Ibaragi, Japan	[52]
NBRC 110837	*Oblongichytrium* sp. 1b	25	Osaka Bay, Japan	[31]
ATCC 28209	*Shizochytrium aggrigatum*	25	North Atlantic Ocean, West Germany	[3]
NBRC 110826	*Thraustochytrium kinnei*	20	Shukugawa River mouth, Hyogo, Japan	[31]
NBRC 110832	*Ulkenia* sp.	20	Osaka Bay, Japan	[31]
NBRC 110846	unidentified thraustochytrid 1	20	Shukugawa River, mouth Hyogo, Japan	[31]
NBRC 110848	unidentified thraustochytrid 2a	25	Shukugawa River, mouth Hyogo, Japan	[31]
NBRC 110856	unidentified thraustochytrid 3c	25	Osaka Bay, Japan	[31]
Diatoms				
NIES 324	*Skeletonema marinoi-dohrnii* complex	25	Osaka Bay, Japan	-
NIES 3712	*Chaetoceros setoensis*	15	Hiroshima Bay, Japan	-

KMPB: Kulturensammlung Mariner Pilze Bremerhaven, Alfred-Wegner-Institut fur Polar und Meeresforschung (Germany), SEK: Laboratory of Systematics and Evolution at Konan University (Japan), ATCC: American Type Culture Collection (USA), NIES: National Institute Environmental Studies (Japan).

### Two-membered culture and cell counting

After 2 weeks of incubating diatom strains in f/2 medium, the diatom cells were harvested, washed, and resuspended in f/2 medium. Zoospores of labyrinthulean strains were inoculated into the diatom cultures. The method to induce zoospores was as follows: vegetative cells were spread on d-GPY agar plates and incubated at 20°C or 25°C for 1 to 2 days. After colonies became visible, seawater was added into the agar plate and incubated for several hours to allow zoospores to be released into the seawater. The *Aplanochytrium* spp. strain ‘unidentified thraustochytrid 1’ (NBRC 110846) did not produce zoospores, so vegetative cells of these strains were inoculated instead. After culturing the vegetative cells in d-GPY liquid medium for 1 week, the cells were harvested, washed, and resuspended in f/2 medium. Fuchs-Rosenthal counting chambers (Minato Medical, Tokyo, Japan) were used for cell counting and observed under BX60 differential interference contrast (DIC) microscope (Olympus, Tokyo, Japan). We defined the cells with intact chloroplasts as living and also defined the cells with shrunk chloroplasts (or empty cells) as dead. The cells of *Aplanochytrium* and *Skeletonema* were continuously counted in single- and two-membered cultures in order to compare the growth process in each culture condition. The cells of *Skeletonema* strain NIES 324 and *Aplanochytrium* strain KMPB N-BA-107 in a single-membered culture in f/2 medium were counted. Also, the cells of *Aplanochytrium* and *Skeletonema* in a two-membered culture in f/2 medium were counted. Cell counts were performed every two days (0, 2, 4, 6, 8, 10 days after starting the culture experiments).

### Two cultures separated by a filter in ‘Beppu Flasks’

To determine the effect of digestive enzymes from *Aplanochytrium* cells in the medium, we established separated cultures in ‘Beppu Flasks’ (Nihon Pall Corporation, Tokyo, Japan) [53], which have two partitions separated by a filter. Using this apparatus, substances such as enzymes that dissolve in the medium can pass through this filter but the organisms cannot. Each partition of the flask contained f/2 medium. *Aplanochytrium* was inoculated into one part and *Skeletonema* was inoculated into the other part. The method of the two-membered culture was as described above. The combinations of *Aplanochytrium* and *Skeletonema* cultured in this system are shown in Result section. The experiments using ‘Beppu Flasks’ confirms whether *Aplanochytrium* cells release components that kill algae, such as digestive enzymes, into sea water or predators that ingest nutrition directly from targeted prey. Statistical analyses were performed using Student’s t-test on the combination of *Aplanochytrium* and *Skeletonema* cell number shown in Fig 8. Significance was accepted at *p* < 0.05.

### Light microscopy

We conducted light microscopy observations using a BX60 differential interference contrast (DIC) microscope (Olympus, Tokyo, Japan) and captured images with an AxioCam HRc camera (Carl Zeiss, Hallbergmoos, Germany). For continuous observations, we used an Axiovert 200 DIC microscope (Carl Zeiss) and captured digital images with an MC170 camera (Leica, Wetzlar, Germany).

### Scanning electron microscopy

The cells were fixed with 2.5% v/v glutaraldehyde and 0.1 M sucrose in 0.1 M cacodylate buffer and were post-fixed with 1.5% OsO_4_. The samples were dehydrated in a graded ethanol series followed by substitution with isoamyl acetate. The samples were successively dried by the CO_2_ critical point method. After applying an OsO_4_ coating, the samples were observed under a JSM-7200F scanning electron microscope (SEM) (JEOL, Tokyo, Japan).

### Analyses of metabarcoding data from Tara Oceans Project

The Tara Oceans project sampled oceanic waters from 2009 to 2013 ([54–55] https://www.embl.de/tara-oceans/). In this study, we used 766 million raw rDNA sequence reads from 334 size-fractionated plankton samples collected from 47 stations. We focused on two depth layers (surface and deep chlorophyll maximum (DCM)). Sequence information was evaluated after dividing the plankton samples into three size fractions (<20, 20–180 and 180–2000 μm). Sequence identification has been described elsewhere [54]. We confirmed the identification of all 351 operational taxonomic units (OTUs) classified in the Labyrinthulea in the Tara Oceans database. All OTUs were added to the aligned sequence data set (Ueda et al. 2015 [31] and Honda et al. 1999[3]), aligned with a profile alignment process using the ClustalX software program [56]. The phylogenetic tree was generated by the neighbor-joining (NJ) method using the distances of the Tamura–Nei model with the pairwise deletion option as the gap treatment in MEGA 7 [57]. The correlations were calculated between aplanochytrids and phototrophs (excluding dinoflagellates), chlorophytes, bacillariophytes, and copepods at the sampling sites where aplanochytrids ranged from most abundant to 30th most abundant. The best fit trendlines were estimated by the method of a least-square linear regression and R-squared values were calculated using with Microsoft Excel software (Microsoft Corporation, USA). Statistical analyses were performed using Student’s t-test between ratio of thraustochytrid in surface and DCM layers.

## Results

### Observations of nutrient intake from diatoms by *Aplanochytrium* cells

**Fig 1:**
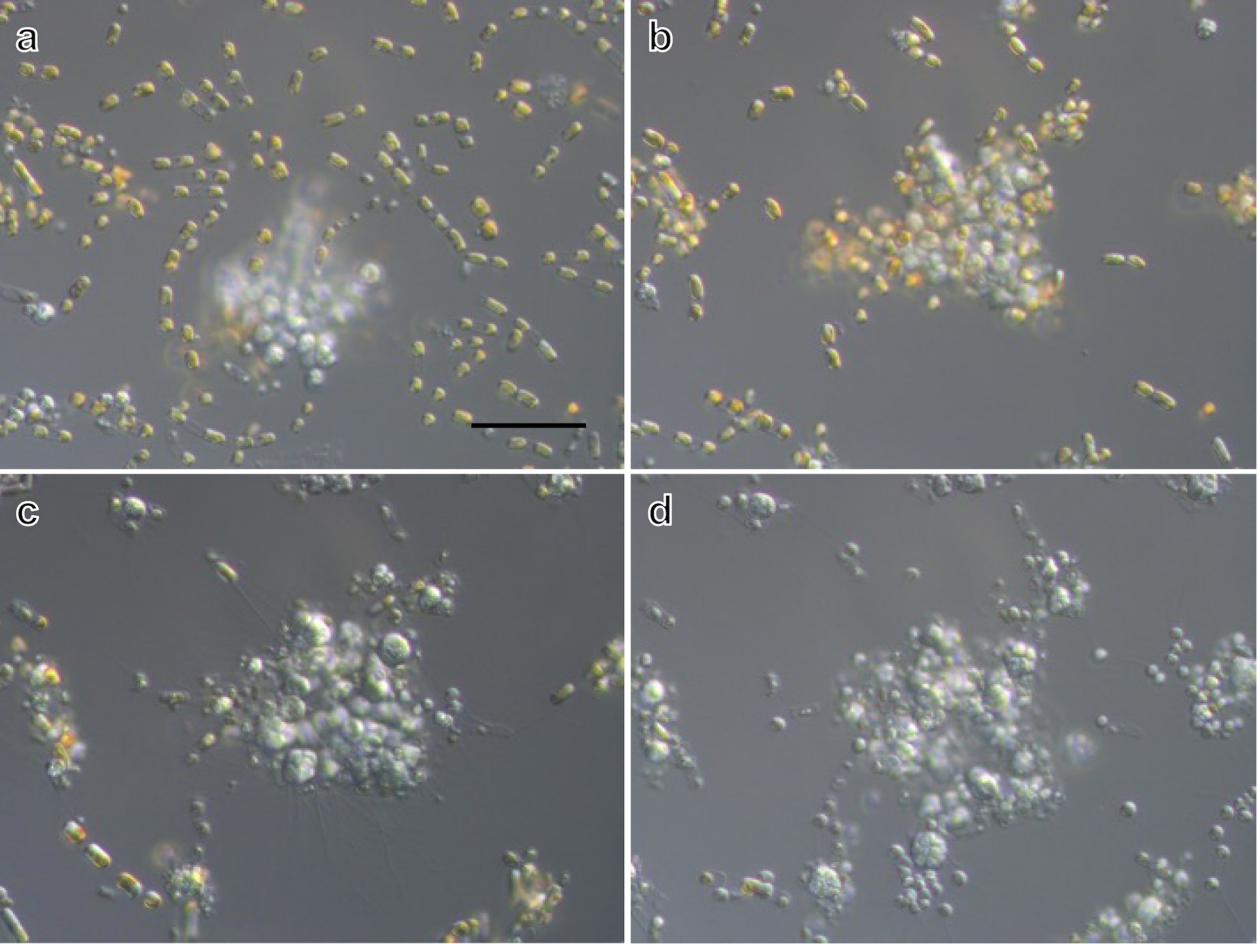
Continuous observations of *Aplanochytrium* and *Skeletonema* in two-membered cultures. (a) Yellow living *Skeletonema* (NIES 324) cells dispersed around small white *Aplanochytrium* (SEK 717) colony, 0 min. (b) Yellow *Skeletonema* cells aggregated around *Aplanochytrium* colony by ectoplasmic nets, 174 min. (c) White *Skeletonema* cells forming aggregates with *Aplanochytrium* cells, 603 min. At this stage, ectoplasmic nets developed radially from *Aplanochytrium* colony. (d) Nutrition intake from *Skeletonema* cells by *Aplanochytrium* almost complete (843 min), accompanied by release of small spherical aplanospores. Scale bar = 50 μm. These continuous observation images can be viewed as a time-lapse video movie (S1 Movie).

**Fig 2:**
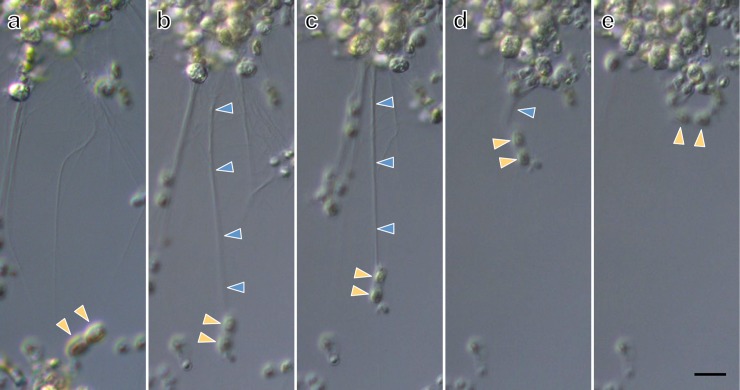
Continuous observation of aggregate formation with *Skeletonema* cells by ectoplasmic nets of *Aplanochytrium*. (a) Two-membered culture of *Aplanochytrium* (SEK 717) and *Skeletonema* (NIES 324). (b) Tip of ectoplasmic nets (blue arrowheads) of *Aplanochytrium* adhering to *Skeletonema* cells (orange arrowheads), 0 min. (c) *Skeletonema* cells (orange arrowheads) are gradually drawn towards aggregate by ectoplasmic nets (blue arrowheads), 45 min. (d–e) *Skeletonema* cells (orange arrowheads) moving near aggregate, 165 and 215 min. Scale bar = 10 μm. These continuous observation images can be viewed as a time-lapse video movie (S2 Movie).

**Fig 3:**
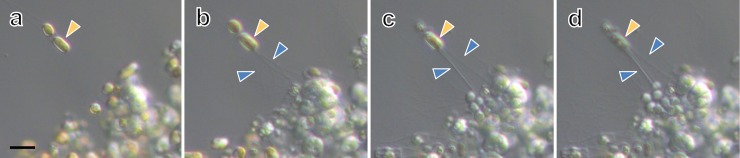
Continuous observation of nutritional intake from *Skeletonema* by *Aplanochytrium* cells via ectoplasmic nets. (a) Two-membered culture of *Aplanochytrium* (SEK 717) and *Skeletonema* (NIES 324). (b) Tip of ectoplasmic nets (blue arrowheads) of *Aplanochytrium* adhering to *Skeletonema* cells (orange arrowheads), 0 min. (c) Ectoplasmic nets increase in size and become more visible, 186 min. (d) In *Skeletonema* cell, chloroplast suddenly shrinks and changes color from yellow to white, 189 min. Scale bar = 10 μm. These continuous observation images can be viewed as a time-lapse video movie (S3 Movie).

**Fig 4:**
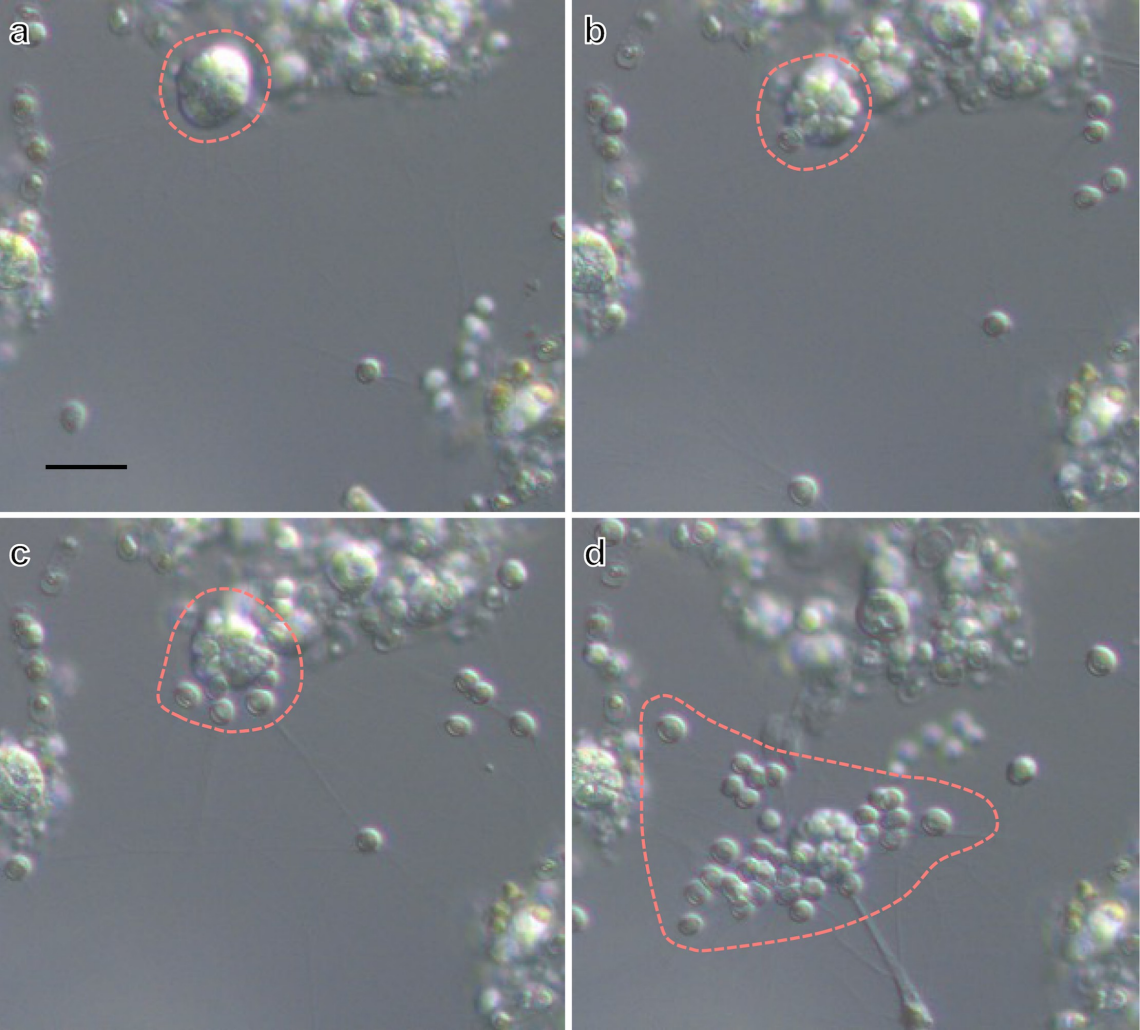
Continuous observation of aplanospore formation. (a) Immature zoosporangium of *Aplanochytrium* (SEK 717), 0 min. (b) Mature sporangium showing cleavage of protoplasm, 28 min. (c) Beginning of aplanospores release, 31 min. (d) Completely released ca. 30 aplanospores, 39 min. Scale bar = 10 μm. These continuous observation images can be viewed as a time-lapse video movie (S4 Movie).

**Fig 5:**
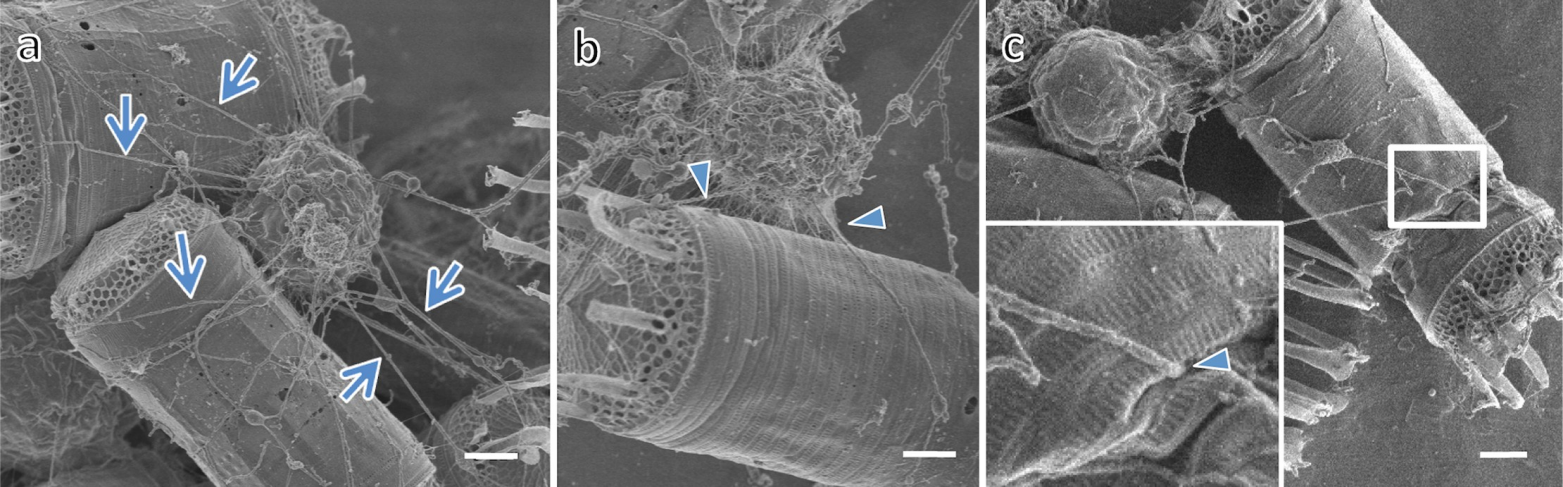
Scanning electron micrographs showing ectoplasmic nets of *Aplanochytrium* cells attacking diatoms. (a–b) *Aplanochytrium* (SEK 717) cells adhering to *Skeletonema* cells via thick web-like (arrows) and fine (arrowheads) ectoplasmic nets. (c) Tip of thick ectoplasmic net penetrating into *Skeletonema* cell (penetration site is shown at high magnification). Scale bar = 1 μm.

*Aplanochytrium* spp. strains have a simple life history, in which the vegetative cells release aplanospores (see [14, 36, 58]). The vegetative cells were spherical with a diameter of 5–10 μm and developed ectoplasmic nets of occasionally more than 50 μm in length (Figs 1–4, S1 Fig). In the two-membered culture of *Aplanochytrium* and *Skeletonema*, the *Aplanochytrium* cells adhered to each other and to *Skeletonema* cells via the ectoplasmic nets and formed aggregates approximately 1000 μm in diameter (Figs 1 and 2). About 2–5 h after the ectoplasmic net attached to a *Skeletonema* cell, the chloroplast suddenly (1–3 min) shrank and the color of the *Skeletonema* cell changed from yellow to white (Figs 1 and 3). When *Aplanochytrium* cells had finished taking nutrition from almost all the *Skeletonema* cells, which had changed from yellow to white, the matured and enlarged vegetative cells of *Aplanochytrium* became sporangia and released aplanospores, which are produced by asexual reproduction (Figs 1 and 4). The number and size of aplanospores were similar to those reported by Moro et al. (2003) [36]. The SEM analysis revealed that the ectoplasmic nets penetrated into the interior of the *Skeletonema* cells through a gap in the silica shell (Fig 5). *Aplanochytrium* cells were also able to ingest nutrition from *Chaetoceros* sp. cells (S2 Fig).

### Comparison of assimilation from diatoms among labyrinthuleans

**Fig 6:**
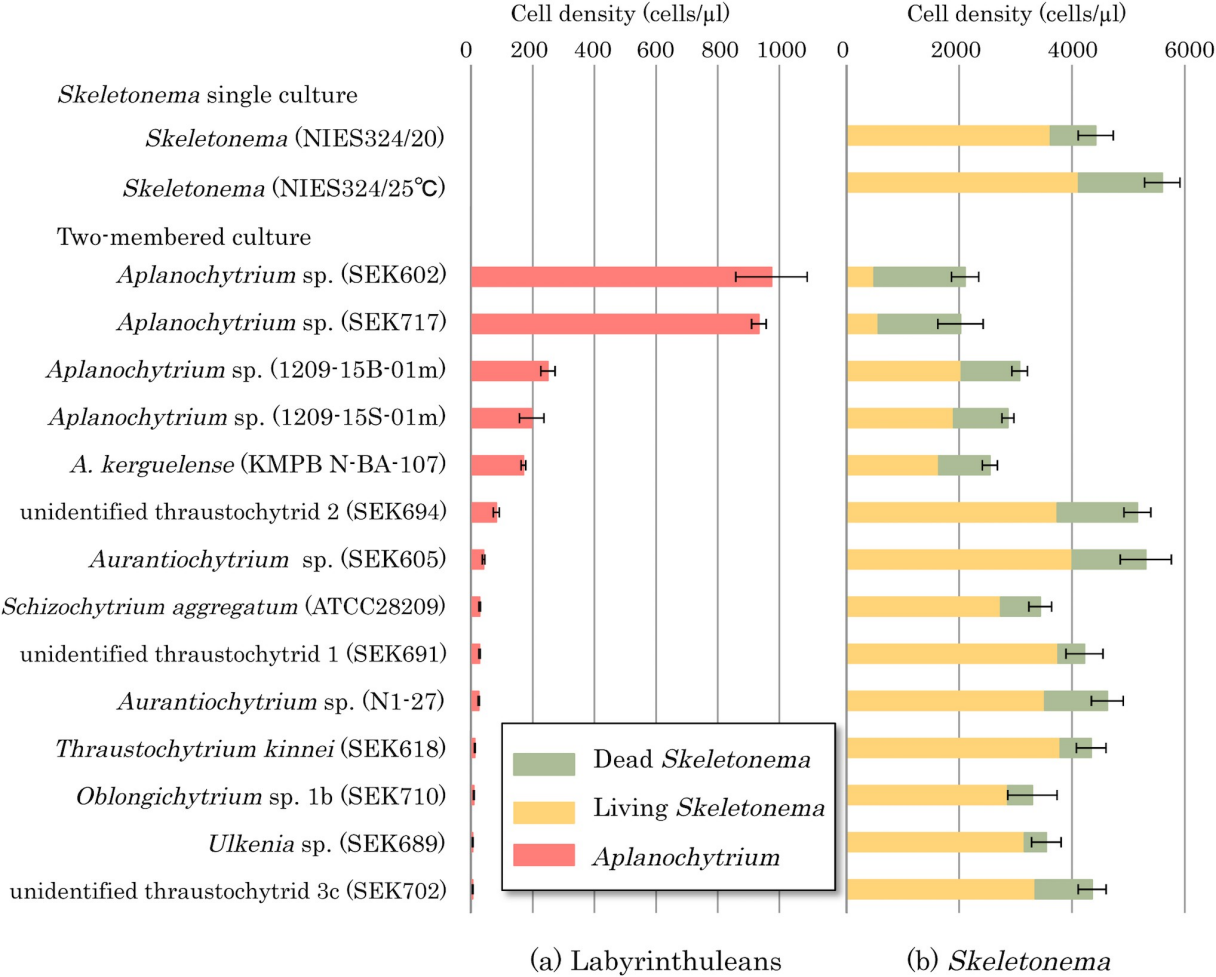
Numbers of labyrinthulean cells and *Skeletonema* living/dead cells in two-membered culture. (a) Cell numbers of labyrinthulean strains. (b) Numbers of living (yellow) and dead (green) *Skeletonema* cells.

The numbers of labyrinthulean cells and *Skeletonema* living/dead cells in the two-membered culture were counted (Fig 6). Previously, it was confirmed that there was almost no increase in the number of labyrinthulean cells in single culture in algal inorganic medium (i.e., f/2 medium). Fig 6 shows the labyrinthulean strains arranged in order of increasing cell density. The cell densities of *Aplanochytrium* sp. strains SEK 602 and 717 were significantly higher than those of other strains. Correspondingly, the abundance of the *Skeletonema* living/dead cells in two-membered cultures were significantly lower with these *Aplanochytrium* sp. strains than with the other strains. The abundance of living *Skeletonema* cells was low, indicating that *Aplanochytrium* sp. strains fed on *Skeletonema* cells faster than the *Skeletonema* cells grew. Three other *Aplanochytrium* spp. strains (KMPB N-BA-107, SEK 754, SEK 758) also showed comparatively high cell densities, and resulted in low abundance of *Skeletonema* cells in two-membered cultures. In these experiments, all *Aplanochytrium* strains obtained nutrition from *Skeletonema* cells via the ectoplasmic nets, but no other tested organisms were able to obtain nutrition from *Skeletonema* cells.

### Continuous cell counting of *Aplanochytrium* and *Skeletonema* in single- and two-membered cultures

**Fig 7:**
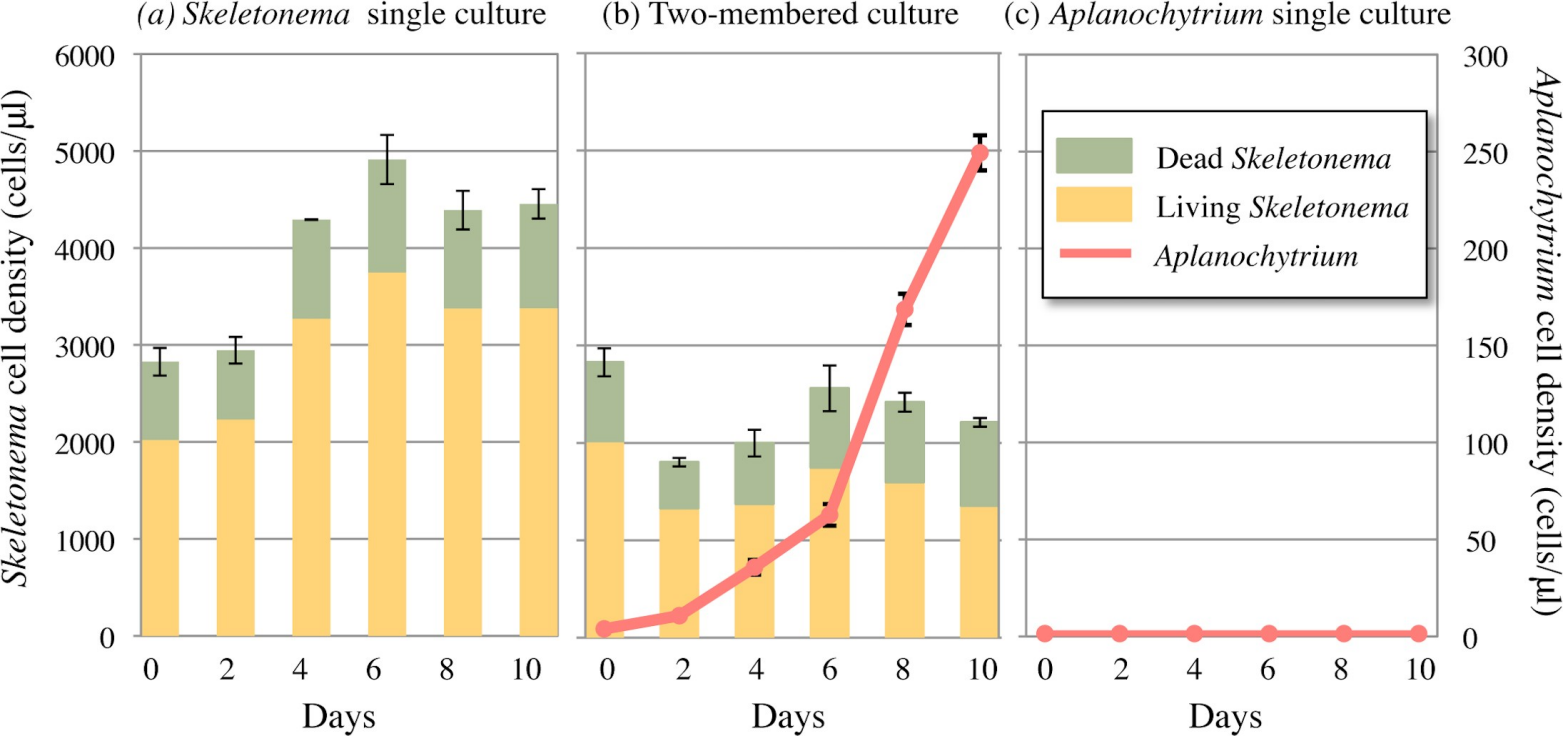
Growth of *Aplanochytrium* and *Skeletonema* in a single- and two-membered cultures. (a) Numbers of living (yellow) and dead (green) *Skeletonema* cells in a single culture. (b) Numbers of living (yellow) and dead (green) *Skeletonema* cells and *Aplanochytrium kerguelense* (KMPB N-BA-107) cells (red line) in two-membered culture. (c) Numbers of *Aplanochytrium* cells (red line) in a single culture.

To explore the predator/prey relationship between *Aplanochytrium* and *Skeletonema*, cells were continuously counted in single- and two-membered cultures. First, the cells of *Skeletonema* strain NIES 324 were counted in a single axenic culture in algal inorganic medium. The cell density increased gradually until the 6^th^ day after the start of culture, and thereafter the number of cells became constant (stationary phase) (Fig 7A). Second, the cells of *Aplanochytrium* strain KMPB N-BA-107 were counted a single axenic culture in algal inorganic medium (Fig 7C). There was no increase in cell density during the 10-day incubation period. In a two-membered culture of *Aplanochytrium* and *Skeletonema* cells, the cell density of *Skeletonema* did not increase, but that of *Aplanochytrium* cells continued to increase logarithmically for 10 days (Fig 7B). These results indicated that *Aplanochytrium* cells grew by absorbing nutrients from *Skeletonema* cells.

### Effect of digestive enzymes from *Aplanochytrium* cells in medium

**Fig 8:**
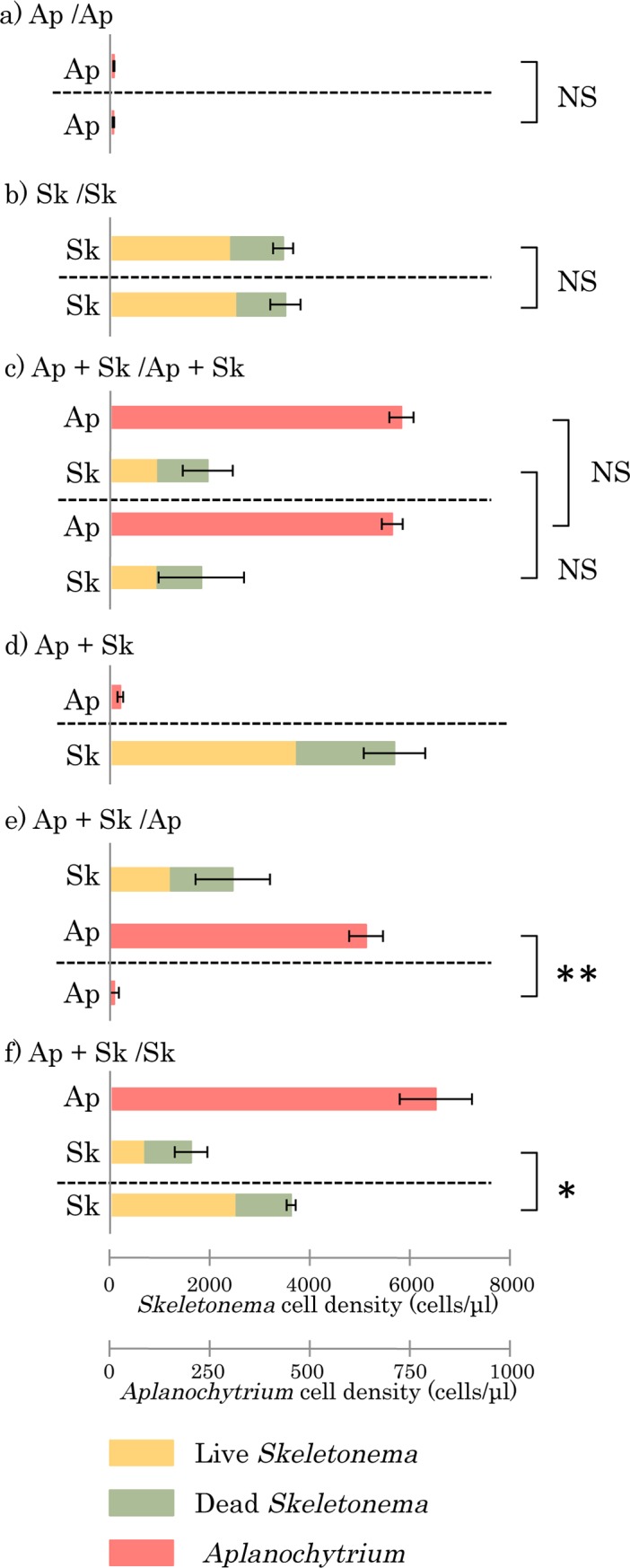
Co-culture experiments to determine effects of digestive enzymes from *Aplanochytrium* on *Skeletonema*. Cell numbers of *Aplanochytrium kerguelense* (Ap, KMPB N-BA-107) and *Skeletonema* (Sk) after 10 days incubation in ‘Beppu Flasks’. (a) *Aplanochytrium* (red) cells cultured both compartments. (b) Numbers of living (yellow) and dead (green) *Skeletonema* cells in both compartments. (c) Two-membered culture of *Aplanochytrium* and *Skeletonema* cells in both compartments. (d) Single culture of *Aplanochytrium* and *Skeletonema* in each compartment. (e) Two-membered culture of *Aplanochytrium* and *Skeletonema* cells in one compartment and single culture of *Aplanochytrium* cells other compartment. (f) Two-membered culture of *Aplanochytrium* and *Skeletonema* cells in one compartment and single culture of *Skeletonema* cells in the other. The single asterisk indicates significant difference (*p* < 0.05) and the double asterisk indicates more significant difference (p < 0.01). NS indicates no significant differences (*p* > 0.05).

As mentioned above, the diatom cells were digested by *Aplanochytrium* cells, suggesting that *Aplanochytrium* cells may produce and secrete digestive enzymes. Therefore, we investigated whether digestive enzymes were released into the medium using the ‘Beppu Flask’ system. The results of control tests are shown in Fig 8A–8C. First, *Aplanochytrium* and *Skeletonema* were cultured in each of the two compartments, As the results in this experiment, *Skeletonema* cells grew while *Aplanochytrium* cells hardly proliferated (Fig 8D). This indicated that *Aplanochytrium* cells did not make soluble secretions of diatoms as nutrients, and did not release digestive enzymes into the medium. Next, *Aplanochytrium* and *Skeletonema* cells were incubated in one compartment as a two-member culture, while *Aplanochytrium* cells were cultured alone in the other compartment. The *Aplanochytrium* cells grew well in the two-membered culture with *Skeletonema* cells, but hardly proliferated in the single culture (Fig 8E). These results suggested that *Aplanochytrium* cells digested the diatoms with degrading enzymes, but the degradants did not disperse in the medium. When *Aplanochytrium* and *Skeletonema* cells were co-cultured in one compartment and *Skeletonema* cells were cultured alone in the other, the *Skeletonema* cells cultured alone grew well, but those co-cultured with *Aplanochytrium* cells did not (Fig 8F). This result indicated that some substances that kill diatoms, such as the digestive enzymes, were not dispersed in the medium from *Aplanochytrium* cells as an influential concentration. Together, these findings indicated that *Aplanochytrium* cells produce decomposing enzymes by contact with diatom cells and can efficiently absorb all the degradation products.

### Relative contributions of *Aplanochytrium* in Tara Oceans Project 18S rDNA metabarcodes

Among 351 TARA Oceans 18S V9 rDNA OTUs classified as Labyrinthulea, 330 OTUs were confirmed as Labyrinthulea by careful phylogenetic analyses and were identified 45, 60, 94 and 131 OTUs as aplanochytrids, oblongichytrids, labyrinthulids and thraustochytrids, respectively (S1 Table).

**Fig 9:**
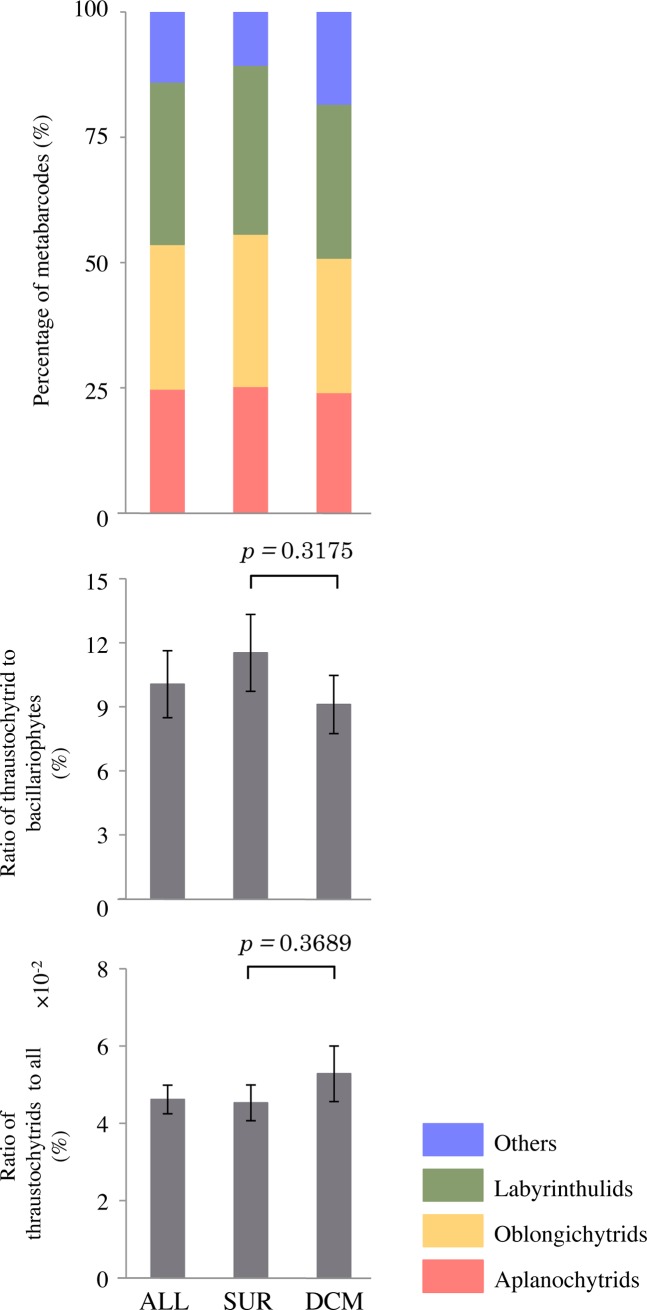
**Relative proportions of main groups of labyrinthuleans (a) and average ratio of labyrinthulean sequences to diatom sequences (b) and to all organisms (c) in surface and DCM waters across all sampling sites.** ALL: surface layer and deep chlorophyll maximum layer, SUR: surface layer, DCM: deep chlorophyll maximum layer. The averaged original data is shown in S3 Fig. The p-values for the pairwise t-test are shown.

We estimated the contributions of the major abundant OTUs in surface and DCM layers (Fig 9A). Three phylogenetic groups (aplanochytrids, oblongichytrids and labyrinthulids) accounted for 80%–90% of total labyrinthulean sequences on average. In the surface layer, aplanochytrids accounted for 25.3% (4.5%–72.5%), oblongichytrids for 30.4% (2.3%–65.4%), and labyrinthulids for 33.7% (3.8%–84.0%) of the total number of sequences identified as Labyrinthulea. In the DCM layer, aplanochytrids accounted for 24.1% (4.1%–56.3%), oblongichytrids accounted for 26.8% (5.2%–51.1%), and labyrinthulids accounted for 30.8% (0.0–60.9%) of the total number of sequences identified as the Labyrinthulea. The average ratios of labyrinthulean sequences to diatoms and to all organisms in surface and DCM layers across all sampling sites are shown in Fig 9B and 9C. The average ratio of labyrinthulean sequences to diatom sequences at each station was about 10% at both depths, while the ratio of labyrinthuleans to all organisms was higher in the DCM than in the surface water.

**Fig 10:**
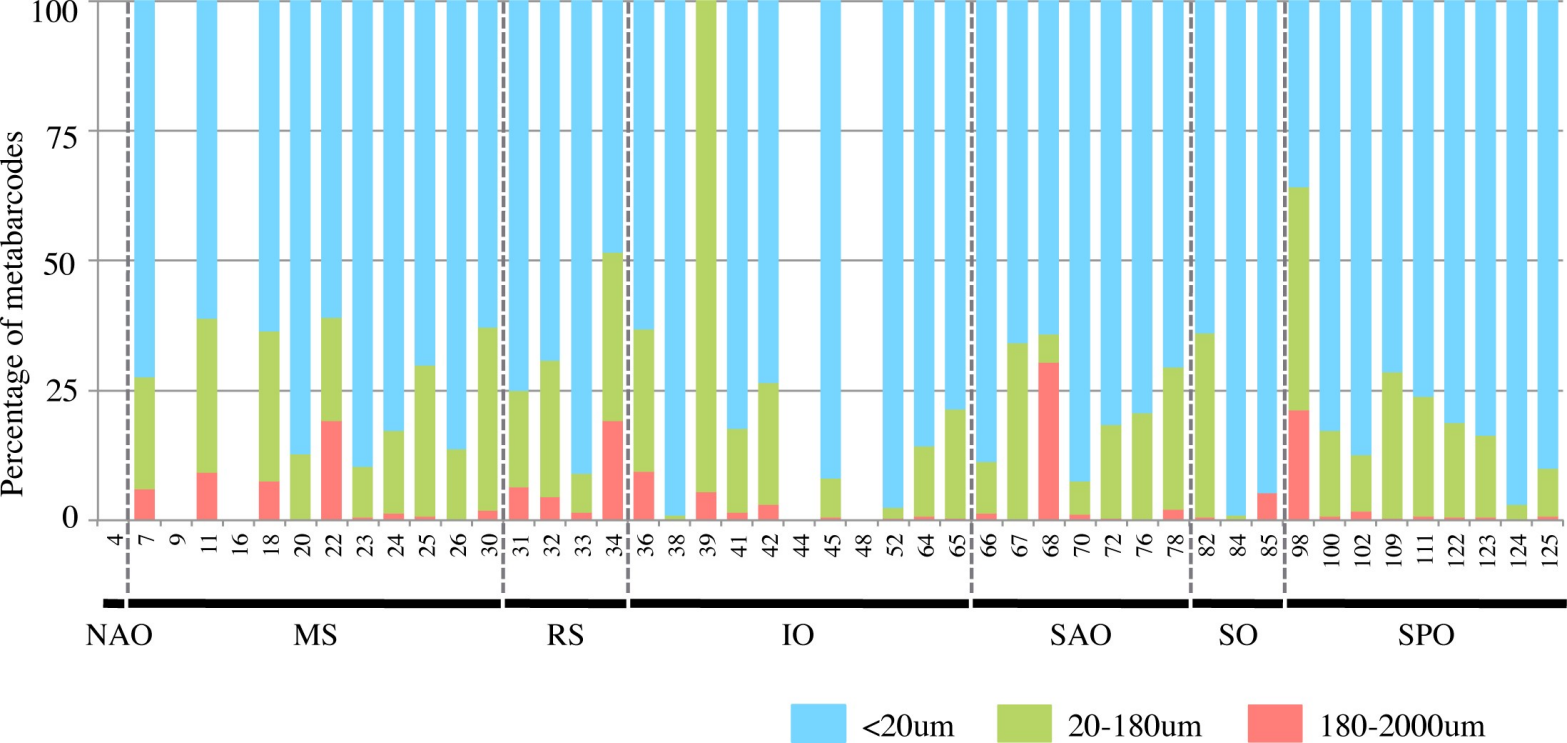
Relative contribution of aplanochytrids in each size fraction of Tara Oceans data set. Ratio of number of sequences in three size fractions (blue: <20 μm, green: 20–180 μm, red: 180–2000 μm) at each sampling site. Note: filter with 200 μm pore size instead of 180 μm was used only at station 82. NAO: North Atlantic Ocean, MS: Mediterranean Sea, RS: Red Sea, IO: Indian Ocean, SAO: South Atlantic Ocean, SO: Southern Ocean, SPO: South Pacific Ocean.

Fig 10 shows the relative contributions of aplanochytrids in the different size fractions in the Tara Oceans V9 data set. The seawater samples from each sampling site were filtered through differently sized filters, and then aplanochytrid sequences were detected in the subsamples with different particle sizes. The average proportions of aplanochytrid sequences out of total sequences in the filtered samples with particle sizes less than 20 μm, 20 to 180 μm, and 180 to 2000 μm were 84.2%, 14.8%, and 2.7%, respectively. A single vegetative cell of *Aplanochytrium* is ca. 5–10 μm, so these results suggested that aplanochytrids formed aggregates with each other and/or with floating matter including digested diatoms, or were eaten by large zooplankton. At 18 sites (43% of the 42 sampling sites), the proportion of aplanochytrids sequences in the subsample with particles of >20 μm exceeded 25%. At three sites, aplanochytrids accounted for more than half of the sequences in the subsample with particles of >20 μm.

**Fig 11:**
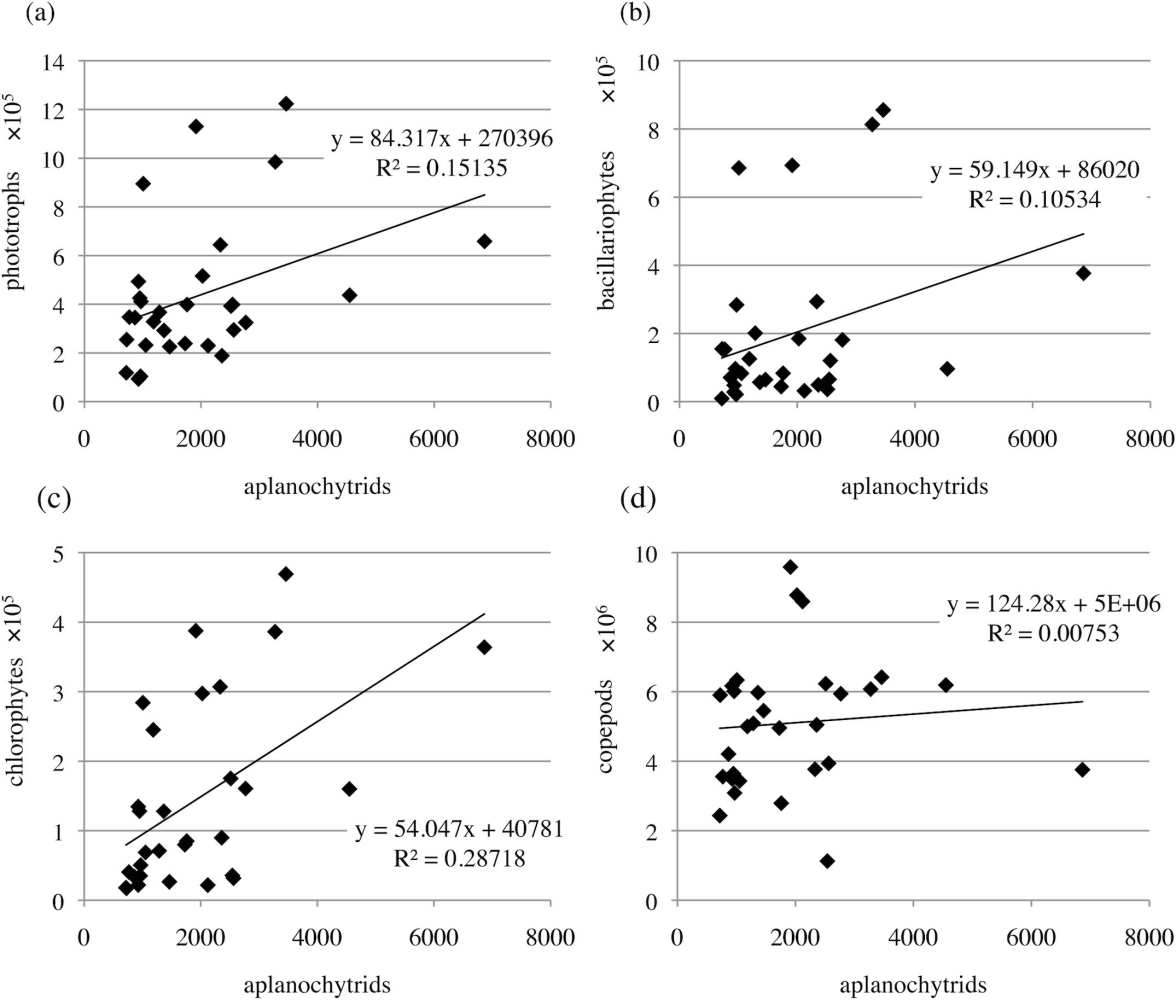
Correlations between *Aplanochytrium* and prey/predators. (a) Phototrophs excluding dinoflagellates. (b) Bacillariophytes. (c) Chlorophytes. (d) Copepods.

We investigated the correlations between aplanochytrids and phototrophs (excluding dinoflagellates), chlorophytes, bacillariophytes, and copepods at the sampling sites where aplanochytrids ranged from most abundant to 30^th^ most abundant (Fig 11). There were positive correlations between aplanochytrids and phototrophs (excluding dinoflagellates), chlorophytes, and bacillariophytes (R^2^ = 0.15, 0.29, and 0.10, respectively). Although aplanochytrids can ingest nutrients from diatoms, it is noteworthy that the highest correlation was between aplanochytrids and chlorophytes. This suggested that aplanochytrids may also ingest nutrients from green algae or their secreted substances. There was almost no correlation between aplanochytrids and copepods (R^2^ = 0.01).

## Discussion

The two-membered culture experiments showed that only *Aplanochytrium* cells consumed nutrients from living *Skeletonema* and *Chaetoceros* diatoms via their ectoplasmic nets. The *Aplanochytrium* cell numbers rapidly increased after releasing multiple aplanospores. The intake the nutrients from diatoms by labyrinthuleans was first reported by Gaertner (1979) [59]. In that study, cells of *Schizochytrium* sp. grew on the surface of the following diatoms: *Thalassiosira nordenskioeldii*, *Chaetoceros* sp. and *Thalassionema nitzschioides*. As the cells of *Schizochytrium* grew, the protoplasm of the diatoms shrank inside the silica shells. Labyrinthuleans adhered to up to 35% of diatom cells in field samples collected from Rosfjord, Norway. Raghukumar (1986) [60] reported that the cells of *Ulkenia visurgensis* adhered to ‘moribund’ diatoms such as *Coscinodiscus*, *Navicula*, *Nitzschia*, *Grammatophora*, and *Melosira*. In our two-membered culture experiments, however, there was little growth of *Schizochytrium aggregatum* and *Ulkenia* sp. with *Skeletonema* diatoms. Taxonomic identification of labyrinthulean cells in environmental samples by microscopic observations is difficult, so species should be confirmed by molecular identification methods. In this study, comparison of cell growth in the two-membered culture experiments suggested that only *Aplanochytrium* strains were able to absorb nutrients from living diatoms.

It was reported that the distribution of thraustochytrids was related to deep chlorophyll maximum layer in the Hawaiian, Arabian and Pacific seas [30, 40, 61–62]. In both of those reports, the cell numbers of thraustochytrids were directly counted after acriflavine dye staining of sulfurylated polysaccharides in the cell wall, so the classification and phylogeny of these thraustochytrids were not clearly shown. Li et al. (2013) [40] showed that 80% of 18S rDNA phylogenetic lineages obtained by sequencing labyrinthulid-specific amplicons aligned with aplanochytrids in Hawaiian sea water. Our experiments showed that *Aplanochytrium* strains are able to absorb nutrients from living diatoms. Therefore, the correlation between cell density of thraustochytrids and chlorophyll value is reasonable. However, it should be noted that recent quantitative PCR and metagenomic analyses of labyrinthuleans in Chinese coastal habitats showed the negative correlation between aplanochytrids and chlorophyll *a* [43].

We explored the dataset generated by the Tara Oceans Project from a wide range of oceanic regions to characterize the diversity patterns of labyrinthuleans on a global scale. At almost all sampling stations, labyrinthulids, oblongichytrids, and aplanochytrids were the major constituent genera and together accounted for about 80% of labyrinthulean organisms. The average proportion of aplanochytrids out of total labyrinthulean organisms at each station was 25.3% and 24.1% in the surface and DCM layers, respectively. In our analyses of the Tara Oceans database, we detected positive correlations between aplanochytrids and phototrophs (excluding dinoflagellates), chlorophytes and bacillariophytes. The average ratio of labyrinthuleans to total diatoms was about 10% (Fig 9). Diatoms contribute around 20% of global primary productivity [51, 63–64]. Thus, labyrinthuleans including aplanochytrids may affect the marine ecosystem.

Aplanochytrids have been detected in the gut and fecal pellets of copepods and salps in the tropical Indian and North Pacific Oceans [34, 37, 39, 65]. Hirai et al. (2018) [39] reported that aplanochytrids, which were recognized by 18S rDNA metagenomic analysis, were possibly the major food source of *Calanus sinicus*, a large copepod common in the coastal waters of the subtropical western North Pacific [66]. The proportion of *Aplanochytrium* was higher in gut contents than in environmental samples, suggesting that *C*. *sinicus* positively selected aplanochytrids as prey [39]. This copepod was shown to actively capture particles with a diameter of 20–50 μm, but few particles smaller than 10 μm [67]. The size of a single vegetative cell of *Aplanochytrium* is 5–10 μm in diameter; however, we observed that *Aplanochytrium* cells adhered to each other and formed aggregates with diatoms, resulting in larger particle sizes. In the environment, labyrinthulean organisms are frequently observed to attach to aggregates [40, 68]. These aggregates may be consumed by *C*. *sinicus* and larger zooplankton.

Labyrinthuleans ingest nutrition via their ectoplasmic nets (e.g., [2, 9, 11–12, 69]). In the present study, we observed that the tip of the ectoplasmic nets of *Aplanochytrium* cells adhered to and penetrated into diatom cells, and then the chloroplasts and cell contents of the diatoms shrank and were absorbed. Because the ectoplasmic nets spread out over a large area forming a fine network to search for prey, they can capture bait at least 50 μm in diameter, even though the single cells are 5–10 μm in diameter.

Our experiments with two cultures separated by a membrane in ‘Beppu Flasks’ showed that *Aplanochytrium* cells could absorb nutrients by directly adhering their ectoplasmic nets to diatoms, without releasing digestive enzymes into the medium. Iwata & Honda (2018) [69] showed that ectoplasmic nets recognize the food source by adhering to it and become thicker, during which secretion of digestive enzymes and absorption of digested substances actively occur. This feeding of labyrinthulean organisms including *Aplanochytrium* is superficially similar to ‘diffusion feeding’, which is known among the heliozoans, foraminiferans, and radiolarians (see [70]). The tentacles of the above-mentioned protists are supported by massive bundles of microtubules and the attached prey is captured in the food vacuole by phagocytosis. In contrast, the ectoplasmic nets consist of actin filaments, and develop a very thin network structure [71–73]. The ectoplasmic net penetrates the diatom cell. The ratio of the volume surrounded by the feeding apparatus to the volume of the cell body is larger in *Aplanochytrium* than in other protozoa. Therefore, feeding of *Aplanochytrium* via its ectoplasmic net is an efficient method to ingest nutrition with a smaller investment than that of diffusion feeding in protists.

In the freshwater environment, the zoosporic true fungi, chytrids, also consume nutrients from phytoplankton, including diatoms, and play an important role as prey for zooplankton in the aquatic ecosystem (see [74]). Chytrids and labyrinthuleans are superficially similar in their morphology at the vegetative stage; that is, both cells are spherical and develop pseudopod-like structures. In *Aplanochytrium*, vegetative cells develop ectoplasmic nets radially around themselves and wait for diatoms to stick to the nets, similar to the strategy of spiders to capture insects in their webs. In chytrids, after zoospores settle on the surface of diatoms, the chytrid cells penetrate and develop rhizoids inside the diatoms to ingest nutrients. In this case, when large phytoplankton that are inedible by zooplankton are infected by chytrids, nutrients within host cells are transferred to zooplankton by grazing on the small edible zoospores of parasitic chytrids, in a pathway known as the ‘Mycoloop’ [74]. Thus, the strategy of nutritional intake using the pseudopod-like structure differs markedly between *Aplanochytrium* and chytrids. However, the nutrients supplied to zooplankton have some similarities. Chytrid zoospores supply poly-unsaturated fatty acids (PUFAs) to zooplankton [75]. Labyrinthuleans produce poly-unsaturated fatty acids, especially docosahexaenoic acid (DHA) [15, 76–81]. Marine fishes contain both DHA and eicosapentaenoic acid (EPA). These fatty acids are important for aquatic ecological processes, and fish in particular require these PUFAs as essential nutrients [82]. In aquaculture, labyrinthuleans have been used to enhance the PUFA content of rotifers and *Artemia* that serve as food for marine fish larvae [83–85]. The survival rate of fish larvae was shown to increase when PUFA-enriched rotifers and *Artemia* were used as food [86–88]. As diatoms accumulate more EPA than DHA, it is easy to predict that diatoms are involved in the accumulation of EPA in fish [89–90]. The present study showed that *Aplanochytrium* spp. are candidates as sources of DHA for zooplankton and fish. Dinoflagellates, haptophytes, cryptophytes, and bacteria are also candidates as sources of DHA [89, 91, 92].

As mentioned above, the consumption of living diatoms by *Aplanochytrium* cells in two-membered culture experiments indicates the possibility there is a newly recognized pathway in the grazing food chain in the marine ecosystem. *Aplanochytrium* spp. are common in the marine environment, from coastal waters to outer oceans, from surface water to deep sea water, and even on phytoplankton in marine snow. However, we have investigated only two diatoms as target organisms nutritionally ingested by *Aplanochytrium* cells, so that it is essential to investigate whether aplanochytrids can ingest nutrients from other algae and protists. Also, it is necessary to clarify whether zooplankton can prey on clusters of *Aplanochytrium* cells and ingest nutrition from them by direct experiments. Furthermore, the ingestion frequency and production efficiency among algae, *Aplanochytrium* and zooplankton should be clarified by the future researches such as experiments using the mesocosms and detailed field studies. By accumulating such knowledge, the roles and impacts of *Aplanochytrium* in the marine ecosystem will be clarified.

## Supporting information

S1 Fig The cells of *Aplanochytrium* spp. strains. (a) *Aplanochytrium* sp. (SEK 717). (b) *Aplanochytrium* sp. (SEK 602). (c) *Aplanochytrium kerguelense* (KMPB N-BA-107). (d–g) Continuous observation of movement of *Aplanochytrium kerguelense* (KMPB N-BA-107), 0, 716, 827 and 865 min respectively.
(TIF)

S2 Fig Continuous observations of *Aplanochytrium* and *Chaetoceros* in two-membered cultures. (a) Two-membered culture of *Aplanochytrium* (SEK 717) and *Chaetoceros* (NIES 3712), 0 min. (b–c) In *Chaetoceros* cell (arrowheads), chloroplast shrinks, 1 and 50 min. Scale bar = 10 μm. These continuous observation images can be viewed as a time-lapse video movie (S5 Movie).
(TIF)

S3 Fig Relative contribution of major groups of thraustochytrid OTUs in different plankton size fractions at Tara Oceans stations. Ratio of thraustochytrid OTUs to bacillariophyte OTUs and all organisms in plankton size fractions in surface (a) and DCM (b) waters. NAO: North Atlantic Ocean, MS: Mediterranean Sea, RS: Red Sea, IO: Indian Ocean, SAO: South Atlantic Ocean, SO: Southern Ocean, SPO: South Pacific Ocean.
(TIF)

S1 Table List of 330 OTUs confirmed as Labyrinthulea by phylogenetic analyses in this study. (DOCX)

S1 Movie *Aplanochytrium* and *Skeletonema* in two-membered cultures. Four images of Fig 1 were extracted from this movie. This time-lapse video corresponds to 14 hours 3 min observation. Scale bar = 50 μm.
(MP4)

S2 Movie Aggregate formation with *Skeletonema* cells by ectoplasmic nets of *Aplanochytrium*. Five images of Fig 2 were extracted from this movie. This time-lapse video corresponds to 4 hours 20 min observation. Scale bar = 10 μm.
(MP4)

S3 Movie Nutritional intake from *Skeletonema* by *Aplanochytrium* cells via ectoplasmic nets. Four images of Fig 3 were extracted from this movie. This time-lapse video corresponds to 6 hours 9 min observation. Scale bar = 10 μm.
(MP4)

S4 Movie Aplanospore formation. Four images of Fig 4 were extracted from this movie. This time-lapse video corresponds to 1 hours 57 min observation. Scale bar = 10 μm.
(MP4)

S5 Movie *Aplanochytrium* (SEK 717) and *Chaetoceros* (NIES 3712) in two-membered cultures. Three images of S2 Fig were extracted from this movie. This time-lapse video corresponds to 16 hours 19 min observation. Scale bar = 50 μm.
(MP4)
