# Supplementary figures and images for: Nutritional intake of Aplanochytrium (Labyrinthulea, Stramenopiles) from living diatoms revealed by culture experiments suggesting the new prey–predator interactions in the grazing food web of the marine ecosystem
Source: PLoS One. 2019 Jan 9;14(1):e0208941. doi: 10.1371/journal.pone.0208941 (PMC6326421; doi:10.1371/journal.pone.0208941)

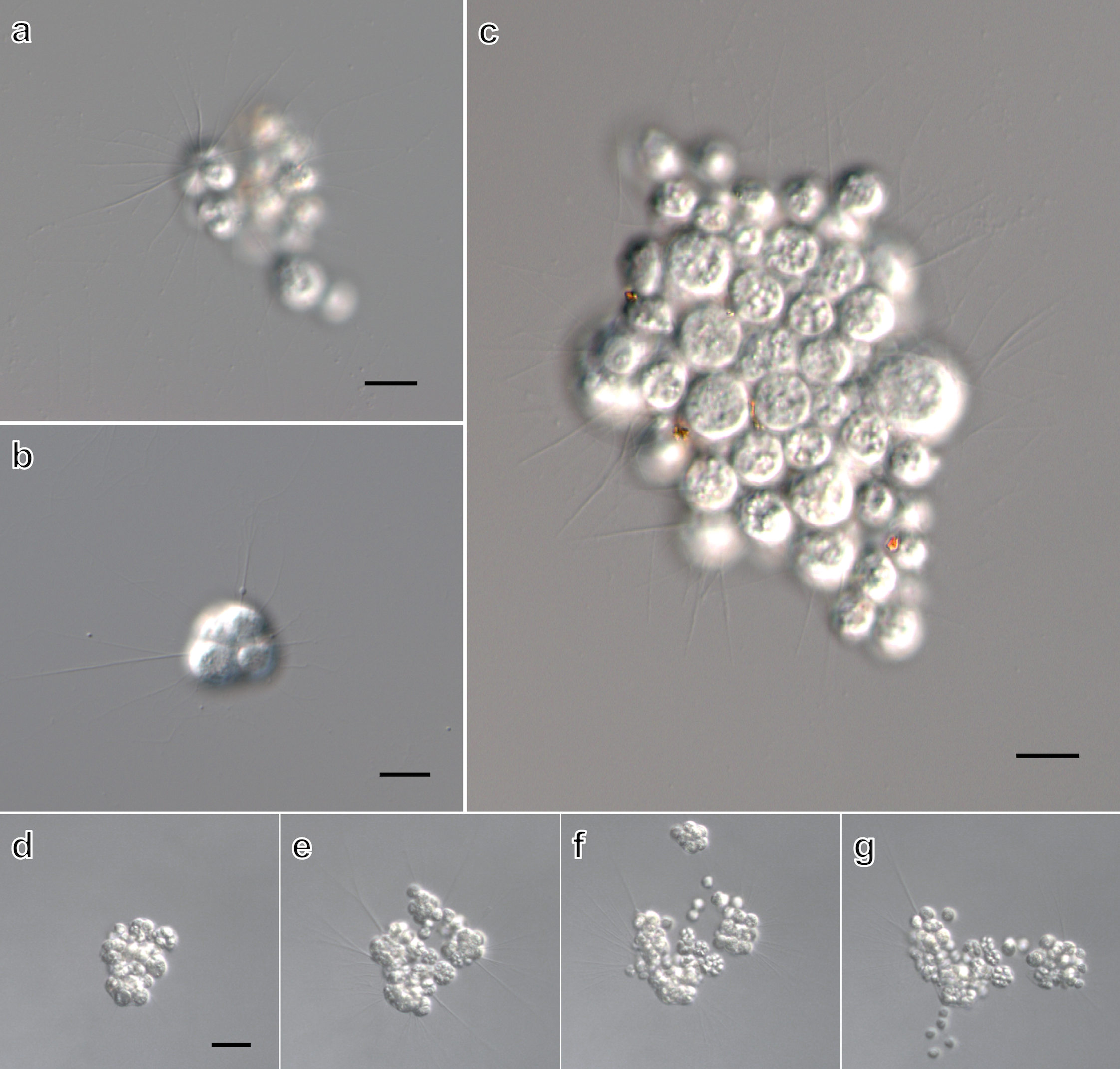

Supplement: S1 Fig — (a) Aplanochytrium sp. (SEK 717). (b) Aplanochytrium sp. (SEK 602). (c) Aplanochytrium kerguelense (KMPB N-BA-107). (d–g) Continuous observation of movement of Aplanochytrium kerguelense (KMPB N-BA-107), 0, 716, 827 and 865 min respectively. (TIF) [file pone.0208941.s001.tif]

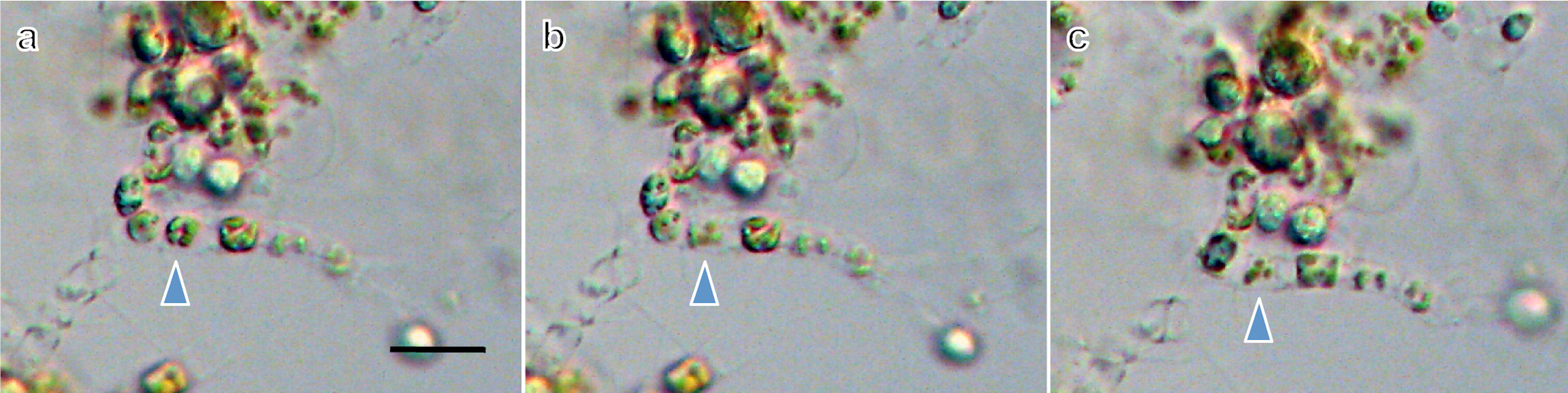

Supplement: S2 Fig — (a) Two-membered culture of Aplanochytrium (SEK 717) and Chaetoceros (NIES 3712), 0 min. (b–c) In Chaetoceros cell (arrowheads), chloroplast shrinks, 1 and 50 min. Scale bar = 10 μm. These continuous observation images can be viewed as a time-lapse video movie (S5 Movie). (TIF) [file pone.0208941.s002.tif]

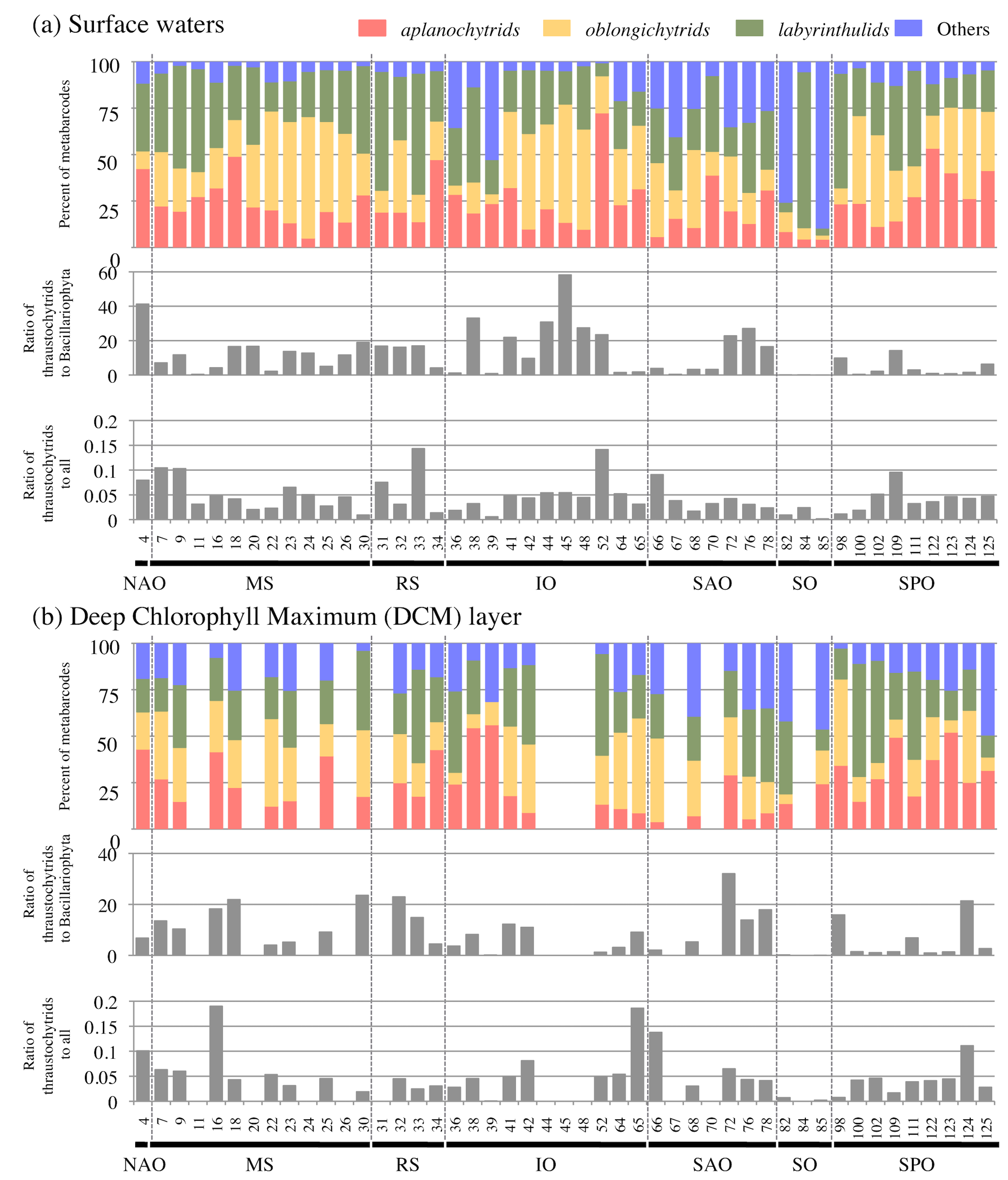

Supplement: S3 Fig — Ratio of thraustochytrid OTUs to bacillariophyte OTUs and all organisms in plankton size fractions in surface (a) and DCM (b) waters. NAO: North Atlantic Ocean, MS: Mediterranean Sea, RS: Red Sea, IO: Indian Ocean, SAO: South Atlantic Ocean, SO: Southern Ocean, SPO: South Pacific Ocean. (TIF) [file pone.0208941.s003.tif]
