# Supplementary material for: Nutritional intake of Aplanochytrium (Labyrinthulea, Stramenopiles) from living diatoms revealed by culture experiments suggesting the new prey–predator interactions in the grazing food web of the marine ecosystem
Source: PLoS One. 2019 Jan 9;14(1):e0208941. doi: 10.1371/journal.pone.0208941 (PMC6326421; doi:10.1371/journal.pone.0208941)
Supplement: S1 Table — (DOCX) [file pone.0208941.s004.docx]

| OTU # | OTU code | | # of reads | Affiliation |
| --- | --- | --- | --- | --- |
| 1 | 4bdd337dc496d45cfd1f62f0cd51b8d1 | 58497 | | aplanochytrids |
| 2 | 1ff80d085fd6b76bc4fd9ae683b2838f | | 1252 | aplanochytrids |
| 3 | 805bf64c779619c75e161f17de75901e | | 478 | aplanochytrids |
| 4 | 962055636af95d80ae08dd1ba4ea360e | | 386 | aplanochytrids |
| 5 | 675324ab17b10b7ac11fa7698e490896 | | 357 | aplanochytrids |
| 6 | 78d823d2d81ae3251e72d9f4ba360c62 | | 357 | aplanochytrids |
| 7 | efd98c9e0af5b20e281b46b2bba2b596 | | 291 | aplanochytrids |
| 8 | ea1607b0181b3181796d9d859024fc24 | | 277 | aplanochytrids |
| 9 | e2db29d2f18ca3678c8ee3420bd5cc16 | | 239 | aplanochytrids |
| 10 | ed2c863bf4bbad1afffb4935233ec363 | | 219 | aplanochytrids |
| 11 | 1f658b5910bc5fb6bf54270e882f1c8c | | 211 | aplanochytrids |
| 12 | fded7939edd5fcd5044d1a1c54323ba7 | | 128 | aplanochytrids |
| 13 | 70c6d58dbe226b9648043ebe2cf17acb | | 118 | aplanochytrids |
| 14 | 44fa63447a971cad23515284a4f6b887 | | 112 | aplanochytrids |
| 15 | 72bc078fcd56964d69479cf05f011317 | | 100 | aplanochytrids |
| 16 | 67f0daa6d6fadb911f4949cf7f20d6f3 | | 74 | aplanochytrids |
| 17 | b4467ceacda741ca130dde56302caa7b | | 48 | aplanochytrids |
| 18 | 9127dfec0dde3bd91e248c833a5c9716 | | 38 | aplanochytrids |
| 19 | 60c67c1aee5f36f7e8a638d35d2f0886 | | 27 | aplanochytrids |
| 20 | f980c4ed1550359a29c5c413bfc23c19 | | 18 | aplanochytrids |
| 21 | 08c3c5adfbbb14e9bf1819465d2e3453 | | 17 | aplanochytrids |
| 22 | 1cf040143d2601123731ec130801d1ff | | 14 | aplanochytrids |
| 23 | bcbffe8f41ab6bd611ab4d25fc14114b | | 13 | aplanochytrids |
| 24 | 751e79dd4462a49a486d3ea4b0b253f7 | | 12 | aplanochytrids |
| 25 | dd60bd20c1aea411263ea40a65bf0b72 | | 12 | aplanochytrids |
| 26 | e68d515d76058ffbf4c57a920f3d529c | | 12 | aplanochytrids |
| 27 | cc23c29e51d515db63f0e577131b8fad | | 9 | aplanochytrids |
| 28 | 982679211a910a1d4e4e080f7932aab3 | | 8 | aplanochytrids |
| 29 | e3b8f0c27eedfa4701af58d4a6eed93f | | 8 | aplanochytrids |
| 30 | 86864f712cd44a19603118ecfd2998f2 | | 7 | aplanochytrids |
| 31 | b7d1e2dcb79f3061d1a25e8ddbd72cd5 | | 7 | aplanochytrids |
| 32 | 0258763ddbf7c5f72409234f8362f5f0 | | 6 | aplanochytrids |
| 33 | 50c8dd31a46b56821889968cc8ec002b | | 5 | aplanochytrids |
| 34 | b23e3f0f0a3f42e4ce99fcacc2c185ae | | 5 | aplanochytrids |
| 35 | 73f0937bae7e99a39fd4114584557b72 | | 4 | aplanochytrids |
| 36 | c49e15bd13b324de6b75100220957c84 | | 4 | aplanochytrids |
| 37 | cfc43f1be533bd672ad6e021f19eafa6 | | 4 | aplanochytrids |
| 38 | 1d1f20432346572a1385e68fe22763ee | | 3 | aplanochytrids |
| 39 | 3424673363b30d95cab284c229352a67 | | 3 | aplanochytrids |
| 40 | 5fe7964b6afb006149af09d0501baea9 | | 3 | aplanochytrids |
| 41 | 608f16d278b2ca80bb9c4553f28452c1 | | 3 | aplanochytrids |
| 42 | 7c11beedd1493d4b45a24881dff67b47 | | 3 | aplanochytrids |
| 43 | 496fcf3a63d99e662e56230ec7653edc | | 2 | aplanochytrids |
| 44 | 1c1bcdc4a786f9a335c41ad71385a3ad | | 1 | aplanochytrids |
| 45 | 5133d7c348cc35e0eae8452f97a9d60a | | 1 | aplanochytrids |
| 46 | 264b99017913aec3e7160a25b071aa71 | | 22018 | labyrinthulea |
| 47 | d3e1bbe4c5a6906e7794e528abbdd40e | | 12083 | labyrinthulea |
| 48 | ed77a91dca6408bedfdbc6805e1f3bc3 | | 10822 | labyrinthulea |
| 49 | a7607486fb7d05b4edf332c228a5b74a | | 8483 | labyrinthulea |
| 50 | 358eca818ab8d85290d9ae4003e5086a | | 7661 | labyrinthulea |
| 51 | 8a849dbbb3a48634e74e00b509617dcc | | 3254 | labyrinthulea |
| 52 | 942f2796eaf505c0ef0bd03ca7da10a4 | | 2916 | labyrinthulea |
| 53 | f513214b061518e07b3c25ffb135af4d | | 2653 | labyrinthulea |
| 54 | 6f17f30cc973169296b137122835dfcb | | 2461 | labyrinthulea |
| 55 | c0a61cd47f4ec8515a128fc8ea071c2e | | 2037 | labyrinthulea |
| 56 | 45f243aa1b74378224828726523899b1 | | 1239 | labyrinthulea |
| 57 | 6db17c966368eb25675bb5cc4706e605 | | 1232 | labyrinthulea |
| 58 | f0e927105b237e2194dd04db415729da | | 1110 | labyrinthulea |
| 59 | 72e2c282f089af75298a19e78a99ee76 | | 833 | labyrinthulea |
| 60 | 4c5b892187c4f5e077d25122484fcf9c | | 584 | labyrinthulea |
| 61 | a8cb5bbd976961b73afffaf50c6efe33 | | 500 | labyrinthulea |
| 62 | b9a2c6a6391be79300b6b89281be2dae | | 287 | labyrinthulea |
| 63 | 051ccbc9d546bae7505e0d05eccb3c16 | | 248 | labyrinthulea |
| 64 | 289ae5ecdf612791883395622131b37e | | 216 | labyrinthulea |
| 65 | da7d67354de72a8bda1fc5aa4c20c815 | | 209 | labyrinthulea |
| 66 | c8824a94780fcfad10667e4860808810 | | 142 | labyrinthulea |
| 67 | 2b8e510c0b1034cc704d2baeb620546e | | 125 | labyrinthulea |
| 68 | a8400533d62ee29bd87bc4e1d5123009 | | 120 | labyrinthulea |
| 69 | d672245b2601f5201513bdfac86aa117 | | 111 | labyrinthulea |
| 70 | 69c33b6162f698b68110ecd292467d81 | | 104 | labyrinthulea |
| 71 | 87f0a7af8806c6d263b86bfde796ab4b | | 94 | labyrinthulea |
| 72 | e5448c565a45c8fde1b430acddef0dbb | | 88 | labyrinthulea |
| 73 | 5f219168f3260aa1c223b122a6e6d11e | | 87 | labyrinthulea |
| 74 | 6eece825b4e0f4a0378380c395a2e6b4 | | 87 | labyrinthulea |
| 75 | a82dcba4d3fba6bcdd3bbb209b70fc93 | | 80 | labyrinthulea |
| 76 | 53ba4cef714818dc2281921efa9efae1 | | 77 | labyrinthulea |
| 77 | c4f0977c771c835b703637331853860e | | 71 | labyrinthulea |
| 78 | 8b5b85cad5c6744a0c25327c263f75a9 | | 64 | labyrinthulea |
| 79 | 5432141d842320b74732c757e598168b | | 63 | labyrinthulea |
| 80 | 35fb282422a76d6d3c9573c025d6b5dd | | 62 | labyrinthulea |
| 81 | d5d726340546001a186dee9e584e5206 | | 48 | labyrinthulea |
| 82 | 347b76d983446186d046124e700ee5f3 | | 44 | labyrinthulea |
| 83 | be216fa40bba28fedb50b41c5c897fa0 | | 38 | labyrinthulea |
| 84 | 0fbdff7e8d4bf380b348a0e0803628a4 | | 33 | labyrinthulea |
| 85 | 930f55cb9beb75cbb9d46932b48bf2d9 | | 32 | labyrinthulea |
| 86 | b14d763c89057214c78c6cc4d66c90e7 | | 32 | labyrinthulea |
| 87 | 228fc867f22b2e9748d9a6f64b3bb502 | | 30 | labyrinthulea |
| 88 | 2fdc9e38619390db697c7a0e55a8e8ec | | 27 | labyrinthulea |
| 89 | e317bdb07b2277d6f38b11cf2847d3bb | | 27 | labyrinthulea |
| 90 | e022bcc682cb23ee1d5ec23cc2d2e642 | | 23 | labyrinthulea |
| 91 | 84290e5ebf2db78c20e78769666a8bc5 | | 21 | labyrinthulea |
| 92 | afeb5a012c84ec4c88a1ef02a89f68b0 | | 21 | labyrinthulea |
| 93 | fe5e6068e622bd103852be3788e61955 | | 20 | labyrinthulea |
| 94 | 1e3c38650ceef171916e4ab299f907d3 | | 19 | labyrinthulea |
| 95 | f54a0835e96a4d52b8b3e55113dfa355 | | 16 | labyrinthulea |
| 96 | f7cdf2c59586003356ff11aa2f3570bd | | 16 | labyrinthulea |
| 97 | c377e49ea7defcaa8f453291ce2efdbc | | 15 | labyrinthulea |
| 98 | b022d677e43538295afe36c8e024d316 | | 14 | labyrinthulea |
| 99 | eab2f1aa1aafab4377ea77ebcabf7510 | | 14 | labyrinthulea |
| 100 | bd8dbbb6f1fea9151fad5b5909802b6c | | 13 | labyrinthulea |
| 101 | 788facf36c5eaa912341190a036dc4ed | | 12 | labyrinthulea |
| 102 | 7b1a1b8dc29cf4e3bf8d282b6dec0add | | 11 | labyrinthulea |
| 103 | ea7e0fbeff1eb69146cf2a36cf6767d6 | | 11 | labyrinthulea |
| 104 | 15f9f04423419e623923acdbc2ac7473 | | 10 | labyrinthulea |
| 105 | ad4e6897084ca7172afbb3195863bade | | 10 | labyrinthulea |
| 106 | 17b576e03b681d00538e4c23c079074c | | 9 | labyrinthulea |
| 107 | de008654a3231fc6753822ee634a1fa6 | | 9 | labyrinthulea |
| 108 | b1eb0b2abf33b7a4797ec09c7bd1d3f8 | | 8 | labyrinthulea |
| 109 | 0cd78adfa4b1d40552b3258294c82852 | | 7 | labyrinthulea |
| 110 | 2eb12d9e73c9c743e1fb8b0af91faddf | | 7 | labyrinthulea |
| 111 | 400eca555a6aac3d3b573873d4129a4f | | 7 | labyrinthulea |
| 112 | 8ffd6e2e184466d38a0a59f6228cadbe | | 7 | labyrinthulea |
| 113 | af5c28866ba12699e24b684ff29b21d7 | | 7 | labyrinthulea |
| 114 | bedc05ab66c4357518ade80de202377c | | 7 | labyrinthulea |
| 115 | 2e78ebbf54b4ed94131e291eb8184952 | | 6 | labyrinthulea |
| 116 | 56fbda71b1f00c698b5fb34f04b2859e | | 6 | labyrinthulea |
| 117 | d647e22c19001a7e02c87aecab529c47 | | 6 | labyrinthulea |
| 118 | 08ca241c7ee0b9e6a692c099f6ae9e36 | | 5 | labyrinthulea |
| 119 | 30ec7d34beeb9dcd9ae4061bf46cd2f0 | | 5 | labyrinthulea |
| 120 | b77b7763bf7f6f108abf76adf052b3ce | | 5 | labyrinthulea |
| 121 | ce36b94ac646863e5efa706d2ec4cc99 | | 5 | labyrinthulea |
| 122 | 49752c0296cef63c93e59891a1dacc7f | | 4 | labyrinthulea |
| 123 | 4abff421370ba938ae6ddbae0f4ea96e | | 4 | labyrinthulea |
| 124 | 9121c4979e950862cf009931b3b172fa | | 4 | labyrinthulea |
| 125 | 9d3e512bb2a7d0e49cbab1b6188f669f | | 4 | labyrinthulea |
| 126 | 17304bceac9c48c100ec5ebec1c84185 | | 3 | labyrinthulea |
| 127 | 522decfee9792f80c2c1ab392fbefedb | | 3 | labyrinthulea |
| 128 | 6a312634e3382571514340be08845a45 | | 3 | labyrinthulea |
| 129 | 8b21d8fa948eb450037afa06974c8151 | | 3 | labyrinthulea |
| 130 | 9851f810f1b5386058ca6c54cbaca6ff | | 3 | labyrinthulea |
| 131 | 9f904d2773dd98609c915bae36303011 | | 3 | labyrinthulea |
| 132 | c202b4322985cbac009f4bea6bb72a0f | | 3 | labyrinthulea |
| 133 | d60408ddc60b861955d2c4a64f8847d2 | | 3 | labyrinthulea |
| 134 | da21275586904767dd086a8638a399cc | | 3 | labyrinthulea |
| 135 | fe2c5474f78915d5d8d2e94a20d25b18 | | 3 | labyrinthulea |
| 136 | 74871db2bb08cd28925cfcbdcd0e114a | | 2 | labyrinthulea |
| 137 | 81c926614a516cca004c57828b9032d4 | | 2 | labyrinthulea |
| 138 | 5534b78f9f1e90d1a2e756fa3991da07 | | 1 | labyrinthulea |
| 139 | 5ac79d7e7ff5e4ecabaf6ecda39302dc | | 1 | labyrinthulea |
| 140 | a17905132d3331696d69a6eec215b165 | | 33670 | oblongichytrids |
| 141 | 0e1868375a02a9fc540f85bb6b80505c | | 26278 | oblongichytrids |
| 142 | bedcd7f6edb5ef006a5dcec216b61684 | | 8169 | oblongichytrids |
| 143 | ed94b9b25767a068ffe0bf91f36207b6 | | 1918 | oblongichytrids |
| 144 | 338a28778065fe0657e66e8153ca0dd8 | | 692 | oblongichytrids |
| 145 | e75485294619fd885c8e48be0caec93f | | 533 | oblongichytrids |
| 146 | e6abbec2c843054bea7762f48e8d444e | | 407 | oblongichytrids |
| 147 | 1211ceba4f9a57ebc9e45854c20b1b6d | | 331 | oblongichytrids |
| 148 | d71cf53db823f269b7aa3a605395eaa8 | | 268 | oblongichytrids |
| 149 | 5bc4cd01621b4b9231f1b148732a8dc7 | | 236 | oblongichytrids |
| 150 | f3746150301cdac46b6c496e50bd7386 | | 203 | oblongichytrids |
| 151 | a2b282ff32610de026a7d9d5cd53c8b9 | | 183 | oblongichytrids |
| 152 | 1987cef92ab9c78f2d95b69f82d8e0b9 | | 173 | oblongichytrids |
| 153 | a9ecdc8c856a80f45d25ab67cef65ed4 | | 162 | oblongichytrids |
| 154 | bceccd928e1f78fb949a52187792c54e | | 104 | oblongichytrids |
| 155 | 9aa288360d4808b5be9adf0fd3c1f5b9 | | 81 | oblongichytrids |
| 156 | bd8d2b26d56b17fb8a2b068b8228cee5 | | 61 | oblongichytrids |
| 157 | e7be89637e3bf5aaaa47b9426e7d4ed1 | | 28 | oblongichytrids |
| 158 | 4ff215d9703ffe03ffeda797a1c864a6 | | 23 | oblongichytrids |
| 159 | 9884306e46612502c6228cc4d126b914 | | 23 | oblongichytrids |
| 160 | 558b0b95520fc8acbfab2e2d91add871 | | 21 | oblongichytrids |
| 161 | 29add138cdb5b8c01dd63a5c6247f8e7 | | 19 | oblongichytrids |
| 162 | a0bde2b89286e7125b5585cadc4282aa | | 19 | oblongichytrids |
| 163 | f0311a7af3a016256e6e3236f4b7f050 | | 17 | oblongichytrids |
| 164 | 5b6fa9ab4d87e485c94b7e06c51900e2 | | 15 | oblongichytrids |
| 165 | b9fe46271e5369aaa215b836db8059e1 | | 12 | oblongichytrids |
| 166 | 634bf915c98e17a80ac8ac86c74a9057 | | 11 | oblongichytrids |
| 167 | a7a087fbe89374eedbc67516528512bf | | 10 | oblongichytrids |
| 168 | 5cc9b8571c28c668ae28d2876724f053 | | 8 | oblongichytrids |
| 169 | 0ad9e32d01ddc60dfcd57d4e9116d6ce | | 7 | oblongichytrids |
| 170 | 7d567fa706e81bd68a2741639b79ed44 | | 7 | oblongichytrids |
| 171 | 2332821f6297a6866bae2b5844074a75 | | 6 | oblongichytrids |
| 172 | 7d340630552cd7464a51e8a495b7cdbd | | 6 | oblongichytrids |
| 173 | be17c93cf1acbd5f50ec261c2bf1552c | | 6 | oblongichytrids |
| 174 | 3f79fb94bf9b55ede32ce12304294ecd | | 5 | oblongichytrids |
| 175 | 570facf3eb2ed66b3c26dbef4cfc2f85 | | 5 | oblongichytrids |
| 176 | b3075691009b51e1c4efd621d21afc7c | | 5 | oblongichytrids |
| 177 | c4336e625cfb0080be64e8103ee912e2 | | 5 | oblongichytrids |
| 178 | d0d6b9c699e9e87f27cbcb495b05e061 | | 5 | oblongichytrids |
| 179 | e9ee8df9254e7e6ba5d91b8e63a3864a | | 5 | oblongichytrids |
| 180 | 1f083c7da57aa74567d70af7af45a009 | | 4 | oblongichytrids |
| 181 | 3085443d7ceee391ec3bbfc22edbbade | | 4 | oblongichytrids |
| 182 | 4a561ae9ef865304c18cdd8fe4991213 | | 4 | oblongichytrids |
| 183 | 84e8ea9092c35b4143ffb3ea77eaa603 | | 4 | oblongichytrids |
| 184 | 9f9fd2706aa7d02d717e49728e83f379 | | 4 | oblongichytrids |
| 185 | bd9395ff3879751cd074a60694bb09e3 | | 4 | oblongichytrids |
| 186 | f9a8a81f66870d0616a1566256ae95ef | | 4 | oblongichytrids |
| 187 | 1618798f865a8a56817b36a4213a85f9 | | 3 | oblongichytrids |
| 188 | 3957d6a1fbcc1240159f7ddc37024090 | | 3 | oblongichytrids |
| 189 | 6aca984d2cc5d36318dcbc812f1b99f4 | | 3 | oblongichytrids |
| 190 | 7942f1d88381bdfbdebe99a10561bc73 | | 3 | oblongichytrids |
| 191 | 7b27093121b182e1a5531581a7f304e6 | | 3 | oblongichytrids |
| 192 | 986fa4e19e0b1edc10222fd65bc7049b | | 3 | oblongichytrids |
| 193 | 9ecd6060d836de6a04c438c93eaf270a | | 3 | oblongichytrids |
| 194 | c2f17c2fa57e5dd4200541449d823cc9 | | 3 | oblongichytrids |
| 195 | c6e45ccf913e289d8c6edbbec968bd71 | | 3 | oblongichytrids |
| 196 | cceaf6f4244957c852268e719756b011 | | 3 | oblongichytrids |
| 197 | f2de9883cf5a01373c4be70c6873fe37 | | 3 | oblongichytrids |
| 198 | 38c4dfc755cc4a3cdd94e941cedbd246 | | 2 | oblongichytrids |
| 199 | 7542f5d7334d1ca30b0163d8c4d0d25a | | 1 | oblongichytrids |
| 200 | 26294f5e4628e4c0fca3f178da8c072d | | 5057 | thraustochytrids |
| 201 | f21685ef9b790b07a2f30b8ee7b1a0f3 | | 4530 | thraustochytrids |
| 202 | 0a5dd510964f46fb23a21da7f1fb8805 | | 4104 | thraustochytrids |
| 203 | 8cdf9b02964933a64d5302bc7cc2ced2 | | 3765 | thraustochytrids |
| 204 | 2ad10341bffd942240975ec138631035 | | 1480 | thraustochytrids |
| 205 | 24c4aac3e9a4df4d59493b61f5079f87 | | 1383 | thraustochytrids |
| 206 | 6ace4910bceb8de9cb633d8b38f802af | | 1258 | thraustochytrids |
| 207 | cd6390e79303cf15bc0a8d10076e9627 | | 1115 | thraustochytrids |
| 208 | 5e5dab6352a31f604a474b0df827ae66 | | 917 | thraustochytrids |
| 209 | aa4038c91c85e43ac89684f2dcb7c8b6 | | 886 | thraustochytrids |
| 210 | 7136f3cec9cb5a8b7baa3e4ce8d1730c | | 804 | thraustochytrids |
| 211 | 1442449d02e004bf3e3e17007d35b29c | | 743 | thraustochytrids |
| 212 | d21d64dff88db2a0c5999ca095f46d0e | | 741 | thraustochytrids |
| 213 | 2bd45d34e946524feff8e6101fa2d501 | | 689 | thraustochytrids |
| 214 | 6e6102cf3173e36f8ce8ad338dd1eb49 | | 657 | thraustochytrids |
| 215 | 956bf69b5499ea4da9207cf54a1d64c4 | | 531 | thraustochytrids |
| 216 | 0ac956c966c39217b25085e9fa150e60 | | 460 | thraustochytrids |
| 217 | 0c7084497edde2fd2b44cce92b07447e | | 389 | thraustochytrids |
| 218 | e479e3ff527fd44c1a12af16a0fa8af0 | | 329 | thraustochytrids |
| 219 | efac8354d64af38ae5b698981e3076ba | | 317 | thraustochytrids |
| 220 | bbd7bb2237db0576145549d075725ca5 | | 311 | thraustochytrids |
| 221 | ce15e72562b272ae14f71a6b1359cec4 | | 309 | thraustochytrids |
| 222 | cd65d99d0f60bac42e5ef8e348b554e6 | | 278 | thraustochytrids |
| 223 | 7fcedae1d51b27ea843d1368912db27b | | 277 | thraustochytrids |
| 224 | 40bec14a1f2f39ecd94f9998d4537362 | | 241 | thraustochytrids |
| 225 | 00fb3357eb0441cdd1e54c19e917daf1 | | 223 | thraustochytrids |
| 226 | 2a40476a358ddcbcfefcff1cca178d36 | | 213 | thraustochytrids |
| 227 | 9a62b71ac68a33e1afe12c60d77b8923 | | 191 | thraustochytrids |
| 228 | a87de179e0c31d9bc5dc526e66c56c3e | | 188 | thraustochytrids |
| 229 | d62e2095fe8dff5049927d0b9c17ed3b | | 183 | thraustochytrids |
| 230 | cbdeb2bb6b6ec92ed9f63a07331da237 | | 179 | thraustochytrids |
| 231 | 9a99b618a516b9d68880697b58de2e8c | | 177 | thraustochytrids |
| 232 | bcd2c47f7fa282bd8e1c1e49d31ccaa4 | | 175 | thraustochytrids |
| 233 | 0b89bbd1fc642fe86251b5366d835415 | | 174 | thraustochytrids |
| 234 | 306df1d946c7a53a39de1c06aef7e1e9 | | 161 | thraustochytrids |
| 235 | 67d41fff32592ec5a19597d45bdc3c4a | | 155 | thraustochytrids |
| 236 | 282ef4a0bf90843336f8778b5cf5b1ee | | 142 | thraustochytrids |
| 237 | a63898738247b4ffc93fd69fe18016f7 | | 106 | thraustochytrids |
| 238 | 7c24390333831951073ad140c7f6a824 | | 102 | thraustochytrids |
| 239 | 55dc61b290ae216af8d74461560730d1 | | 101 | thraustochytrids |
| 240 | 6d7a62acbcf4aabe317a05b91e78c333 | | 84 | thraustochytrids |
| 241 | a6dcf2c25d92ce1790cd2d42124708cb | | 82 | thraustochytrids |
| 242 | 2c601f0ba97aa7b0eb0d0eb9003f979a | | 81 | thraustochytrids |
| 243 | 17a469f3c6ce1b0d6323d891f252aeab | | 79 | thraustochytrids |
| 244 | bd9fcd968388d2005ae3eedc5de387ee | | 72 | thraustochytrids |
| 245 | e0347448c702e04ee200c4074ee6a52f | | 68 | thraustochytrids |
| 246 | 355f68a64d7f9ab83b6530f282aa8124 | | 64 | thraustochytrids |
| 247 | 8550f4732b1efb4cefbd392469e428a3 | | 63 | thraustochytrids |
| 248 | 9329832be86642566fd56ffeb151cd5f | | 55 | thraustochytrids |
| 249 | 3f573ed06f0c693799cd7d1af20486b2 | | 52 | thraustochytrids |
| 250 | 06bf8cde5f6ab29e929fb141a16eb32a | | 51 | thraustochytrids |
| 251 | 3dc43adfd02ecfc780728967e361e8b2 | | 49 | thraustochytrids |
| 252 | c911080e2d16b7964ce90598d2cf0b23 | | 35 | thraustochytrids |
| 253 | 49f4f797f88d87a5da9946e612b1e8c0 | | 33 | thraustochytrids |
| 254 | 03063b95d45f9247148b06e429208f7d | | 32 | thraustochytrids |
| 255 | 4db6c85271102e12ab84d973a58a958c | | 30 | thraustochytrids |
| 256 | 59159c8782196051707cdceddae1f171 | | 30 | thraustochytrids |
| 257 | e39dbd80302fdecb8f0e05da74fe7933 | | 28 | thraustochytrids |
| 258 | a8a0a6ff46bc23963aeb7db544958df1 | | 27 | thraustochytrids |
| 259 | c2905e0dfc38871c1c28a4de871c12e4 | | 26 | thraustochytrids |
| 260 | bd5c31a072560b9f64d88ad244726260 | | 25 | thraustochytrids |
| 261 | 64fc6964f6ebae1a1077603095e51962 | | 23 | thraustochytrids |
| 262 | 56ccee4a62b66c59a960b865ac78832e | | 22 | thraustochytrids |
| 263 | 6679a8cabb1992747e80ab8457930da9 | | 19 | thraustochytrids |
| 264 | fd31193897f456b3c2d87fb3bd8ddecc | | 18 | thraustochytrids |
| 265 | 13508454540d62b63732c9382d46767b | | 16 | thraustochytrids |
| 266 | 6093e61f4291fad285b28bb9594b8e07 | | 15 | thraustochytrids |
| 267 | 216275b3605d0325507387626ee14ae8 | | 14 | thraustochytrids |
| 268 | 38b70e4e935fb2f94c62e415487b7b63 | | 14 | thraustochytrids |
| 269 | 2cd155d681d618fc5cd3e282f160ec2b | | 13 | thraustochytrids |
| 270 | d6690d7eccdc4133f93c297ba39dec32 | | 13 | thraustochytrids |
| 271 | 6f3e3a30d0295c42dd34dc247da52866 | | 12 | thraustochytrids |
| 272 | 2bf010eac293bea132777efaedfadd33 | | 11 | thraustochytrids |
| 273 | b6a69bf1a3ef5743b64b90f2666abb27 | | 11 | thraustochytrids |
| 274 | 20dfc92addffe3a7b9902e33582bfa29 | | 10 | thraustochytrids |
| 275 | 220e2b42c57b220b9baf1c25aec71367 | | 10 | thraustochytrids |
| 276 | 274f53cc9853b636e17b78f07aa337c0 | | 10 | thraustochytrids |
| 277 | 5bc93f63389065957de48d367a73774f | | 10 | thraustochytrids |
| 278 | 81f3a6e8e2d63ddc50e15d3dc12ec72a | | 10 | thraustochytrids |
| 279 | e696e3ac85389d1f8fbf1d8b900efb2c | | 10 | thraustochytrids |
| 280 | f765c9fb6bfc171b3c43932c4187d2f8 | | 10 | thraustochytrids |
| 281 | 2685737d5518272e46d2c93e7d29165e | | 9 | thraustochytrids |
| 282 | 3583d2dedc48e5df7070846b82f579b8 | | 9 | thraustochytrids |
| 283 | c5b83c2adcdbd7295d46ea0807a44625 | | 9 | thraustochytrids |
| 284 | c8266d99083f42332ec5e293ea814a23 | | 9 | thraustochytrids |
| 285 | ce43a2d0aa00a1a2998eebcf90d72fe3 | | 9 | thraustochytrids |
| 286 | 2fbed860fa8777065b0b8dccc4e06607 | | 8 | thraustochytrids |
| 287 | 44981815848c59279dfa58fbeae1b56d | | 8 | thraustochytrids |
| 288 | 5fdf050d643cb9832c9cac717b0c4c84 | | 8 | thraustochytrids |
| 289 | 960e329b247488e17b050f7819d3af87 | | 8 | thraustochytrids |
| 290 | b2158de2410e236c22dee84789023fc5 | | 8 | thraustochytrids |
| 291 | 02d9d420997fb05bca066de981c273eb | | 7 | thraustochytrids |
| 292 | 0e3284c463b497396ac21f27f963cad1 | | 7 | thraustochytrids |
| 293 | 7454b5a766973da8222e256e2d925c22 | | 7 | thraustochytrids |
| 294 | 952205d58d0460c0f9852b37d6a8eafd | | 7 | thraustochytrids |
| 295 | a3540c5d3780c042ee5584fd259925b4 | | 7 | thraustochytrids |
| 296 | 87ab5b9cb3749b1b54b813059408eef5 | | 6 | thraustochytrids |
| 297 | 9a2779ce17f5e83cd310e0c6087bca5f | | 6 | thraustochytrids |
| 298 | bef8488549a5a9582035a0dc0ddf7737 | | 6 | thraustochytrids |
| 299 | da2d7f908e1f4dad4413dfa225e3268e | | 6 | thraustochytrids |
| 300 | 3e48576f56f13fc00fa74451b60b8ca8 | | 5 | thraustochytrids |
| 301 | 3e61328489e786e9e85767351db0c1e9 | | 5 | thraustochytrids |
| 302 | 6232e36d676f5ac5eaad06b0c1545483 | | 5 | thraustochytrids |
| 303 | 7a7d17c31fef2499b690513d826ef81a | | 5 | thraustochytrids |
| 304 | bc372a29ec3be4dc90d5826b2abc6452 | | 5 | thraustochytrids |
| 305 | d26d849dc766b5a05ba5927376177136 | | 5 | thraustochytrids |
| 306 | 04e8f8e48dd73fb4a8a4646d53524529 | | 4 | thraustochytrids |
| 307 | 1283f0573f01d5b31d4a059e3fce082b | | 4 | thraustochytrids |
| 308 | 13ee9c93351e0a0f18c1d477225c4109 | | 4 | thraustochytrids |
| 309 | 40c799f626f435eb9336cae6c231252e | | 4 | thraustochytrids |
| 310 | 67c5467620994f30239c8ca477299d76 | | 4 | thraustochytrids |
| 311 | b6be3c846ec003b6842ff13714224569 | | 4 | thraustochytrids |
| 312 | c624f4bd3eea28bb75b2776b04b4aeb3 | | 4 | thraustochytrids |
| 313 | d107dc310b00255801296312707de479 | | 4 | thraustochytrids |
| 314 | f2440e21e1ec5a6f567e4574c75d4412 | | 4 | thraustochytrids |
| 315 | fb728a1b5cc79550016057e5111dbc92 | | 4 | thraustochytrids |
| 316 | fbf0de3e03b5a3e9af9f08c7f61084d5 | | 4 | thraustochytrids |
| 317 | 034b3a01ce753a6b5077d5ba4d0e30e2 | | 3 | thraustochytrids |
| 318 | 71c04b0e609817082860f0e0afe723c0 | | 3 | thraustochytrids |
| 319 | 7ba6cefb929607d2be9ec5863c47eaa4 | | 3 | thraustochytrids |
| 320 | 837fb45dae5fe73022e49da92d8afe3c | | 3 | thraustochytrids |
| 321 | 865e373a5a3562d4a0d43ec019862869 | | 3 | thraustochytrids |
| 322 | a0d8495b0da1939d9b75a4eb6034983a | | 3 | thraustochytrids |
| 323 | aab8158023c06ee8fab349a09af00bfe | | 3 | thraustochytrids |
| 324 | b284b537e5f0a06e77ea887e50e3dc4d | | 3 | thraustochytrids |
| 325 | ba0c81ca3445cc9045640f9b3fc44139 | | 3 | thraustochytrids |
| 326 | cae47611b83973230342edf09a4e8b41 | | 3 | thraustochytrids |
| 327 | e990191b928a55444649d39c20534d68 | | 3 | thraustochytrids |
| 328 | 70efec7fd7df96541418f5a3f320cf94 | | 2 | thraustochytrids |
| 329 | a5dbf2b4aa8ab16dcf1362cc13b4daff | | 2 | thraustochytrids |
| 330 | 0c920aa401c1acdf564908e0ab47f73a | | 1 | thraustochytrids |
| 331 | 7fce6b15e0a3597a33580a339a4e2510 | | 40342 | not labyrinthuleans |
| 332 | 5b91de13ee62b5812cd2d994738cb421 | | 9259 | not labyrinthuleans |
| 333 | affc5ab3f32df94b6de32808ff7d3dfb | | 4600 | not labyrinthuleans |
| 334 | b27810161d5d9c509e15fb56e2ca3d03 | | 3370 | not labyrinthuleans |
| 335 | 5092933412db8bb2d805e5bbad0cd73c | | 1845 | not labyrinthuleans |
| 336 | 3ffa8be3e6299c976466af643cd29736 | | 1301 | not labyrinthuleans |
| 337 | 9c407c800629238c3f28e5d78229a537 | | 492 | not labyrinthuleans |
| 338 | c5591ba6cfdd4eb533dc6b7e318c7be0 | | 367 | not labyrinthuleans |
| 339 | 03be54cd2f9c52247845211b4fdf9af1 | | 238 | not labyrinthuleans |
| 340 | 5cf871643bbd6fa93220af51d56d84cc | | 178 | not labyrinthuleans |
| 341 | 247fbc7ade72d69c64b335c2e569bc7d | | 78 | not labyrinthuleans |
| 342 | 9767c0f097f9a709c00cb20333994277 | | 52 | not labyrinthuleans |
| 343 | bc2d8d7a2f7bff6f2e23e71fedff2073 | | 26 | not labyrinthuleans |
| 344 | 099f5a59508fa46e8708c441f9baf744 | | 11 | not labyrinthuleans |
| 345 | 8a343bf5bad6a7c51dd5616802a4c425 | | 6 | not labyrinthuleans |
| 346 | bd90457e41be44426f91df5140fb41b8 | | 4 | not labyrinthuleans |
| 347 | 3f55850c19b128d120ee11e5e9376858 | | 3 | not labyrinthuleans |
| 348 | 7c0886ee20cdc2e757878c4c048cd52c | | 3 | not labyrinthuleans |
| 349 | b1db039ef1775f5cc711574f8b2554ad | | 3 | not labyrinthuleans |
| 350 | 29114b9f1d5dc06b78c96b126c938040 | | 2 | not labyrinthuleans |
| 351 | c641ae534588ea8a1239fa5637b0890e | | 2 | not labyrinthuleans |
